# Multiplex proteomics identifies novel CSF and plasma biomarkers of early Alzheimer’s disease

**DOI:** 10.1186/s40478-019-0795-2

**Published:** 2019-11-06

**Authors:** Christopher D. Whelan, Niklas Mattsson, Michael W. Nagle, Swetha Vijayaraghavan, Craig Hyde, Shorena Janelidze, Erik Stomrud, Julie Lee, Lori Fitz, Tarek A. Samad, Gayathri Ramaswamy, Richard A. Margolin, Anders Malarstig, Oskar Hansson

**Affiliations:** 1 Research and Early Development (RED), Biogen Inc., Cambridge, MA 02139 USA; 2 Clinical Memory Research Unit, Department of Clinical Sciences, Lund University, Lund, Sweden; 3 Wallenberg Center for Molecular Medicine, Lund University, Lund, Sweden; 4 Pfizer Worldwide Research & Development, Cambridge, MA 02139 USA; 5 Department of Medicine, Karolinska Institutet, Vetenskapsvagen 10, 171 76 Stockholm, Sweden; 6 Rare and Neurologic Disease Research, Sanofi Research and Development, Framingham, MA 01701 USA; 7 CNS Research Solutions LLC, Cambridge, MA 02139 USA; 8 Memory Clinic, Skåne University Hospital, SE-20502 Malmö, Sweden

**Keywords:** Alzheimer’s disease, Mild cognitive impairment, Biomarker, Proteomics, Inflammation, Apoptosis, Angiogenesis

## Abstract

To date, the development of disease-modifying therapies for Alzheimer’s disease (AD) has largely focused on the removal of amyloid beta Aβ fragments from the CNS. Proteomic profiling of patient fluids may help identify novel therapeutic targets and biomarkers associated with AD pathology. Here, we applied the Olink™ ProSeek immunoassay to measure 270 CSF and plasma proteins across 415 Aβ- negative cognitively normal individuals (Aβ- CN), 142 Aβ-positive CN (Aβ+ CN), 50 Aβ- mild cognitive impairment (MCI) patients, 75 Aβ+ MCI patients, and 161 Aβ+ AD patients from the Swedish BioFINDER study. A validation cohort included 59 Aβ- CN, 23 Aβ- + CN, 44 Aβ- MCI and 53 Aβ+ MCI. To compare protein concentrations in patients versus controls, we applied multiple linear regressions adjusting for age, gender, medications, smoking and mean subject-level protein concentration, and corrected findings for false discovery rate (FDR, *q* < 0.05). We identified, and replicated, altered levels of ten CSF proteins in Aβ+ individuals, including CHIT1, SMOC2, MMP-10, LDLR, CD200, EIF4EBP1, ALCAM, RGMB, tPA and STAMBP (− 0.14 < *d* < 1.16; *q* < 0.05). We also identified and replicated alterations of six plasma proteins in Aβ+ individuals OSM, MMP-9, HAGH, CD200, AXIN1, and uPA (− 0.77 < *d* < 1.28; *q* < 0.05). Multiple analytes associated with cognitive performance and cortical thickness (*q* < 0.05). Plasma biomarkers could distinguish AD dementia (AUC = 0.94, 95% CI = 0.87–0.98) and prodromal AD (AUC = 0.78, 95% CI = 0.68–0.87) from CN. These findings reemphasize the contributions of immune markers, phospholipids, angiogenic proteins and other biomarkers downstream of, and potentially orthogonal to, Aβ- and tau in AD, and identify candidate biomarkers for earlier detection of neurodegeneration.

**Electronic supplementary material:**

The online version of this article (10.1186/s40478-019-0795-2) contains supplementary material, which is available to authorized users.

## Introduction

Alzheimer’s disease (AD) is the most common cause of dementia, affecting one in 10 people aged 65 years or older [3]. To date, the development of disease-modifying treatments for AD has largely targeted one of its pathological hallmarks, amyloid beta (Aβ), and to a lesser extent tau, with notably high failure rates in clinical trials [12]. Molecular markers such as amyloid PET, cerebrospinal fluid (CSF) Aβ, and CSF tau are frequently employed in the clinical diagnosis of AD, and biomarkers are increasingly recognized as integral components of the drug development pipeline [10]. However, recent evidence from human genetics [45, 52], neuroimaging [14], and in-vivo modeling [9] has strongly implicated additional disease mechanisms in AD, not reflected by the established Aβ and tau biomarkers. These potential pathophysiological processes, such as inflammation [14, 52], membrane phospholipid dysregulation [62], and neurovascular disruption [45], are less frequently targeted, and remain less well understood [22].

Proteomics, the analysis of proteins in different bodily tissues and fluids, can be used to study myriad pathways putatively affected in AD in-vivo, before the onset of overt neurodegeneration, thus potentially elucidating important disease mechanisms and informing future pharmacotherapeutic trials. In CSF, certain proteomic assays can reflect underlying brain pathology [48], and thus, may be useful for pharmacokinetic monitoring, diagnosis, and stratification [5]. However, lumbar puncture is invasive. Blood may serve as a more accessible body fluid; however, many blood-based molecular profiling studies of AD have been restricted by small sample sizes, methodological variability, and suboptimal diagnostic sensitivity [28, 38]. Additionally, with the exception of certain blood biomarkers, such as plasma Aβ [39] and plasma tau [28], few studies have directly explored the relationship between CSF proteins and their blood-based analogs in the same cohort; thus, the extent to which peripheral molecular changes accurately reflect CNS dynamics has yet to be characterized at large scale.

Here, we sought to understand the contributions of proteins downstream of, and potentially orthogonal to, Aβ and tau across the preclinical, prodromal, and dementia stages of AD. We measured a diverse panel of 270 proteins in the CSF and plasma of up to 1022 individuals, using a validated, highly sensitive and specific immunoassay [4]. We identified, and replicated, evidence of differential regulation in proteins related to innate and adaptive immunity (CD200, CHIT1, MMP-9, MMP-10, oncostatin-M, STAMBP), membrane phospholipids (LDLR), axon guidance, cell adhesion and differentiation (ALCAM, RGMB, ROBO2), ischemic injury (uPA, tPA, SMOC2), mTOR and Wnt/β-catenin signaling (AXIN1, EIF4EBP1), and glucose metabolism (HAGH) in early and later-stage AD. Multiple proteins associated with baseline cortical thickness and cognitive performance. Approximately half of all assayed proteins in CSF showed modest correlations with their analogs in plasma, and a combination of plasma analytes could differentiate AD from non-AD with high accuracy. Thus, our findings reemphasize the importance of targeting pathways beyond Aβ and tau in AD, and identify several candidate biomarkers for early detection of brain pathology, pending additional validation.

## Materials and methods

### Standard protocol approvals, registrations, and patient consents

The study was approved by the Regional Ethics Committee in Lund, Sweden. Written informed consent was collected from all participants.

### Study participants

Participants were recruited from southern Sweden between 2009 and 2014 as part of the prospective and longitudinal BioFINDER study (www.biofinder.se). A total of 872 participants assessed at the Memory Clinic in Malmö were recruited to the discovery cohort, including 565 cognitively normal elderly participants (CN), 131 patients with mild cognitive impairment (MCI), and 176 patients with dementia due to AD (see Table 1). An additional 179 participants assessed at the Memory Clinic in Lund were recruited to the replication cohort, including 82 elderly participants who presented with memory complaints but were clinically determined to be cognitively normal, and 97 MCI patients (see Additional file 1: Table S6).

**Table 1:** Demographic and clinical data for the Memory Malmö ‘discovery’ cohort

	Control Aβ-	Control Aβ+	MCI- Aβ-	MCI- Aβ+	AD (Aβ+)	P-value^a^ (group differences)
Sample size (n)	415	142	50	75	161	
Sex (F/M)	256/159	100/42	20/30	33/42	104/57	0.000061
Mean age in years (SD)	71.7 (5.21)	72.99 (4.78)	68.8 (5.12)	72.29 (4.93)	74.58 (7.37)	1.676 × 10−10
MMSE mean (SD)	29.11 (0.9)	29.02 (0.83)	27.3 (1.68)	26.56 (1.79)	21.47 (3.9)	9.4022 × 10−213
*APOE* (1 or 2 ɛ4 alleles)	22.89%	57.75%	20%	74.67%	66.46%	7.0724 × 10−32
Anti-inflammatory drugs	9.64%	7.75%	4.00%	9.33%	6.21%	0.52
Platelet inhibitor drugs	16.39%	17.61%	38.00%	33.33%	29.19%	0.000016
Antidepressive drugs	6.75%	7.04%	32.00%	16.00%	22.98%	1.3371 × 10−10
Lipid-lowering drugs	26.02%	30.99%	42.00%	37.33%	29.81%	0.07
Antihypertensive/cardioprotective drugs	41.69%	49.30%	54%	52%	54.04%	0.04
Current smoker	9.4%	2.82%	8%	5.33%	9.94%	0.08
Mean Aβ42 in pg/ml (SD)	752 (253)	423 (175)	628 (223)	280 (90)	305 (132)	6.3545 × 10− 119
Mean Aβ40 in pg/ml (SD)	5847 (2042)	6566 (2373)	4956 (2045)	5057 (1612)	5470 (2179)	4.9837 × 10−8
Aβ42/40 ratio - log2 transformed (SD)	2.05 (0.17)	2.75 (0.28)	2.05 (0.17)	2.89 (0.28)	2.9 (0.29)	7.8397 × 10−253
Mean total tau (SD)	292 (89)	432 (163)	295 (110)	515 (181)	649 (221)	
Mean phospho-tau (SD)	37 (13)	66 (35)	40 (17)	105 (46)	123 (47)	

Demographics are provided for participants who were included in the final proteomics analysis after quality assessment (see Methods)

^a^To assess group differences we used a test of independence (Chi-square) for categorical variables and ANOVA for continuous variables

Cognitively normal elderly participants were included as study controls if they (i) were aged 60–80 years, (ii) had Mini-mental State Examination (MMSE) scores of 28–30 at their initial screening visit, (iii) lacked symptoms of cognitive impairment, as assessed by a physician, and (iv) did not fulfill the criteria for MCI or dementia. Participants were excluded from the control group if they (i) refused lumbar puncture, or if they presented with (i) a significant neurological or psychiatric disease (iii) current alcohol or substance misuse, or (iv) a systematic illness preventing them from participating in the study.

Patients with MCI were recruited from a larger cohort of non-demented outpatients with cognitive symptoms; the inclusion criteria for this cohort included (i) age 60–80 years, (ii) initial presentation with a complaint related to memory, executive, visuo-spatial, language praxis, or psychomotor function, (iii) an MMSE score between 24 and 30, (iv) significant impairment in at least one cognitive domain (most often memory) according to an assessment by an experienced neuropsychologist [26] and (iv) essentially preserved activities of daily living. Exclusion criteria included (i) fulfillment of the criteria for any dementia disorder, (ii) cognitive impairment that could be definitively explained by another condition, (iii) a systemic illness preventing them from participating in the study, and (iv) refusal to undergo lumbar puncture or neuropsychological assessment.

AD dementia patients were classified using the criteria for probable AD, as defined by NINCDS-ADRDA [29].

Participants were grouped based on a combination of their clinical diagnosis (AD, MCI, or CN) and CSF Aβ pattern, based on combined Aβ40 and Aβ40 assays (Eurimmun, Germany). Individuals with a CSF Aβ42/Aβ40 ratio ≥ 0.1 were considered amyloid-negative controls (Aβ- CN) or patients with MCI not due to AD (Aβ- MCI), and individuals with a ratio ≤0.1 were considered amyloid-positive cognitively normal participants (Aβ+ CN), or patients with MCI due to AD (Aβ+ MCI) [20, 41]. All patients with dementia due to AD had pathological CSF ratios ≥0.1. The CSF Aβ42/Aβ40 ratio was used instead of CSF Aβ42 alone, as this ratio has a greater concordance with amyloid PET findings [20, 41]. Aβ- MCI patients were included in the study for comparison with Aβ+ patients, since proteins showing evidence of differential regulation across Ab-positive and Ab-negative groups could potentially implicate processes orthogonal to Aβ deposition.

Following quality control, the Memory Malmö discovery cohort consisted of 415 Aβ- CN participants, 142 Aβ+ CN participants, 50 Aβ- MCI patients, 75 Aβ+ MCI patients, and 161 AD patients (see Table 1 for participant demographics). The Memory Lund replication cohort consisted of 59 Aβ- CN participants, 23 Aβ+ CN participants, 44 Aβ- MCI patients, and 53 Aβ+ MCI patients (see Additional file 1: Table S6 for participant demographics).

### Magnetic resonance imaging

MRI data were collected from 303 healthy elderly controls and 112 MCI patients from the Memory Malmö cohort, using a 3 Tesla Siemens® Trio scanner equipped with a standard 12-channel head coil. Hippocampal volume and cortical thickness estimates were measured using FreeSurfer v5.3 (https://surfer/nmr.mgh.harvard.edu). A composite AD ‘signature cortical thickness’ measurement was constructed by calculating mean surface-area adjusted thickness across the entorhinal, inferior temporal, middle temporal, and fusiform cortices bilaterally [17]. Hippocampal volume measures were adjusted for total intracranial volume. Full details of MRI processing are described elsewhere [40].

### Plasma and CSF sampling

Plasma and lumbar CSF samples were collected from non-fasting participants during the ’subjects’ baseline BioFINDER visit. and analyzed following a standardized protocol [40]. After collection, samples were centrifuged (2000 g, + 4 °C, 10 min), aliquoted into 1 ml polypropylene tubes (Sarstedt AG & Co., Nümbrecht, Germany), and stored at -80 °C. Prior to proteomic screening, all plasma and CSF samples underwent one freeze-thaw cycle, and were further aliquoted into 200L Lobind tubes (Eppendorf Nordic A/S, Denmark).

### Protein quantification

Protein concentrations were quantified using the validated, highly sensitive and specific ProSeek multiplex immunoassay, developed by Olink Proteomics (Uppsala, Sweden) [4]. Three commercially available ProSeek® Multiplex panels were used to measure the concentrations of 270 proteins in human plasma and CSF (Additional file 1: Table S1). Protein measurements were conducted using Proximity Extension Assay (PEA) technology, following the manufacturer’s protocol [4]. Briefly, antigens were incubated with pairs of antibodies containing DNA oligonucleotides bound to each of the 270 proteins to be measured. A template for hybridization and extension was generated by oligonucleotides in close proximity, and pre-amplification was performed using polymerase chain reaction (PCR). Following digestion of residual primers, specific primers were digested on a quantitative real-time PCR chip (Dynamic Array IFC; Fluidigm Biomark) using a Biomark HD Instrument. Protein quantities were produced as normalized protein expression (NPX) values on the log_2_ scale.

### Patient-level quality control

Samples from 1051 participants (872 discovery samples, 179 replication samples) were randomized and assayed in parallel on 54 ProSeek plates in plasma and 55 ProSeek plates in CSF. Four internal controls were added to each sample to monitor assay performance and the quality of individual samples, and intensity normalization was implemented to minimize technical variation between plates. The quality of each sample was assessed by evaluating deviation from the median value of its internal control; samples that deviated less than 0.3 NPX from the median passed quality assessment.

### Protein-level quality control

Proteins detected in more than 70% of samples were considered sufficient to allow statistical analysis using standard regression models. Only proteins with less than 20% of values below the reported lower limit of quantification (LLQ) were analyzed quantitatively. In these instances, we used the actual raw extended values (under LLQ), provided by OLINK, to impute best-guess values. A histogram was made of each such protein to verify a continuous distribution of values across the LLQ threshold when using these extended values.

Principal component analyses (PCAs) were generated on the NPX values and visually inspected to identify possible outliers and to evaluate the consistency of plasma and CSF data.

### Statistical analysis

All statistical analyses and data processing were conducted using R version 3.4.4 and IBM SPSS Statistics for Windows 22.0. All univariate linear regressions and mixed-effects models conducted in the discovery cohort were adjusted for False Discovery Rate (FDR) at an FDR-adjusted *p*-value (i.e. *q* value) threshold of 0.05, using the *p.adjust* function in R. For proteins surviving FDR adjustment in the discovery cohort, unadjusted *p*-values were inspected in the replication cohort.

#### Univariate linear regressions

Differential regulation of proteins was assessed between the cognitively normal, Aβ- elderly CN and; (i) cognitively normal, Aβ+ elderly individuals, (ii) Aβ- MCI patients, (iii) Aβ+ MCI patients, and (iv) patients with dementia due to AD, using multiple linear regressions via the *lm* function in R, where clinical grouping was the predictor of interest, and protein concentration in plasma or CSF was the outcome measure. Age, gender, current smoking status, medications (including platelet inhibitors, antidepressants, anti-inflammatory, lipid-lowering, antihypertensive, and cardioprotective drugs), and cross-subject mean protein concentration, collectively referred to as ‘standard covariates’, were included in all models. For brevity, comparisons between clinical groups (AD versus Aβ+ MCI, Aβ+ MCI versus Aβ+ CN, AD versus Aβ+ CN) were omitted from the main text, but included in the Additional file 1: (see Additional file 1: Note 1 and Tables S23-S28).

#### Multivariate LASSO regression

We employed multivariable modeling to identify subsets of proteins that could accurately discriminate cognitively normal healthy individuals from cognitively normal Aβ+ individuals, MCI, and AD-dementia. Our model evaluated several different proteins simultaneously, using the least absolute shrinkage and selection operator method (LASSO; [55], implemented via the *glmnet* package in R. We separately tested models to predict Aβ- MCI, Aβ+ MCI, and AD-dementia patients, versus Aβ- controls, adjusting for age and gender. The LASSO regularization parameter lambda was selected by 10-fold cross-validation using the *cv.glmnet* function, to protect against overfitting. Results from the LASSO models are presented as the mean and 95% CI of the cross-validated area under the receiver operating characteristics curves (AUC). In a supplementary analysis, we employed a more complex LASSO model which adjusted for the standard covariates (Additional file 1: Note 2).

#### Associations with clinical and neuroimaging endpoints

Associations between circulating proteins and Mini-Mental State Examination (MMSE; collected for all participants) and Clinical Dementia Rating Scale Sum of Boxes (CDR-SB; collected for healthy elderly and MCI) were assessed using multiple linear regressions in R, where baseline MMSE or CDR-SB score was the determinant of interest, and protein concentration was the outcome measure, adjusting for the standard covariates.

Associations between circulating proteins, hippocampal volume, and signature cortical thickness were assessed using multiple linear regressions in *R*, again adjusting for the standard covariates.

#### Within- and between-fluid correlations

Within-fluid correlations (CSF-to-CSF, plasma-to-plasma) and between-fluid correlations (CSF-to-plasma) were assessed across all proteins using a simple Pearson correlation test in R.

## Results

### 190 CSF proteins and 250 plasma proteins were analyzed across 843 discovery samples and 179 replication samples

Due to technical issues, no data were recorded from the BDNF and CCL22 assays. An additional 68 CSF proteins and 18 plasma proteins were excluded due to low detection rates (< 70%; Additional file 1: Table S1). Thus, our final statistical analyses included 190 proteins that were detected in > 70% of CSF samples, and 250 proteins that were detected in > 70% of plasma samples.

Of the 1051 samples, 29 failed due to missingness of 10% or more analytes in CSF. 31 samples (including the aforementioned 29 samples) had missingness rates of 10% or more in plasma. The remaining samples had no more than five missing values (representing 1.8% of all analytes). A single healthy elderly participant was identified as an outlier in plasma, due to a covariate-adjusted PCA value more than 3.5 standard deviations from the centroid (Additional file 1: Figs. S1-S8). Thus, of the initial 1051 participants, 1022 were eligible for plasma analysis, and 1019 were eligible for CSF analysis.

### CSF levels of CHIT1, MMP-10, SMOC2, and ezrin were increased in AD and Aβ-positive MCI

**Fig. 1:**
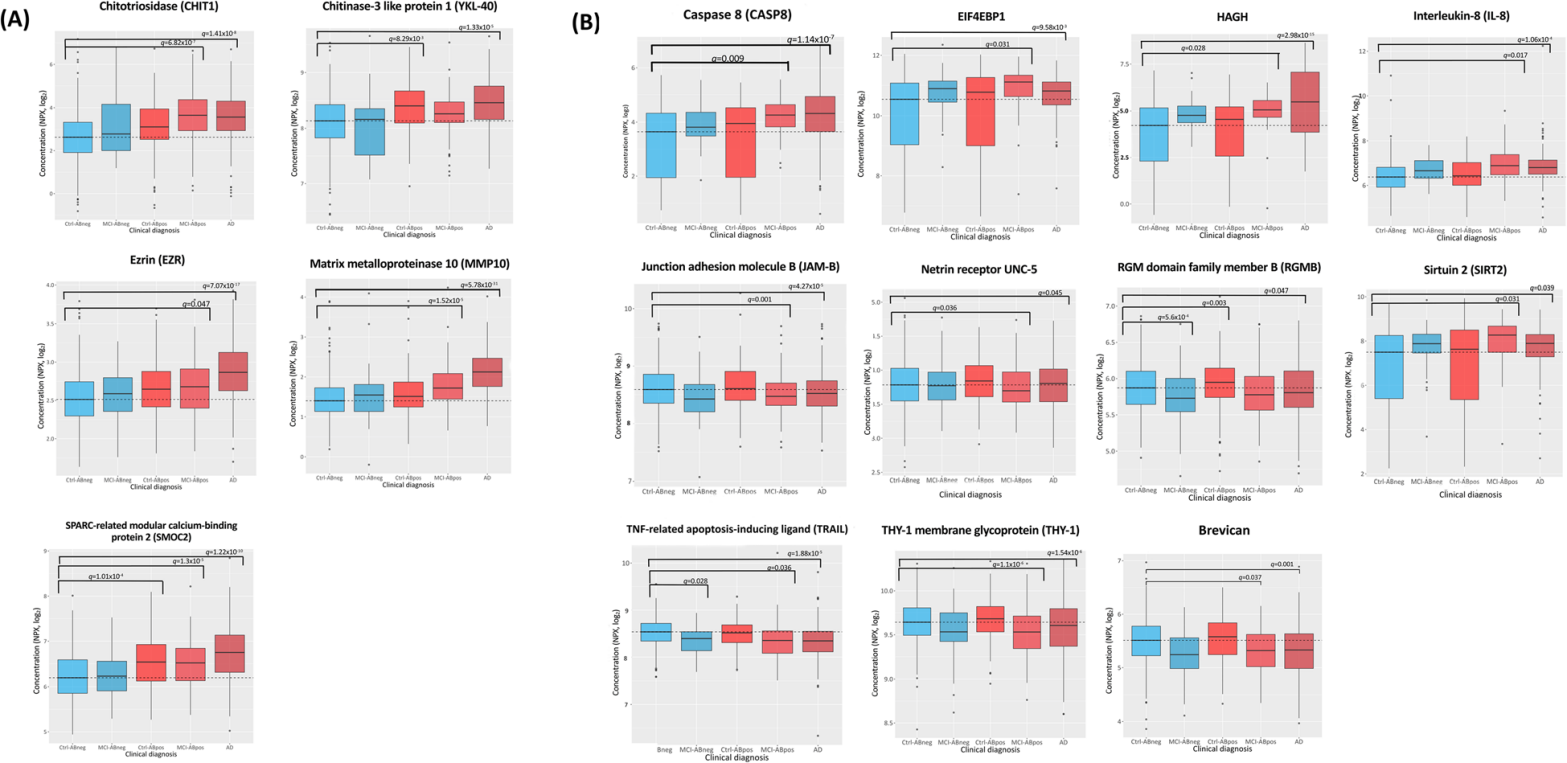
Proteins showing evidence of differential regulation in two or more Aβ+ groups, in a cerebrospinal fluid (CSF) and b plasma. Dashed line represents the average protein concentration of the Aβ- cognitively normal group (i.e. study controls). Aβ- groups are coloured in blue; Aβ+ groups are coloured in red. Ctrl-ABneg = Aβ- controls; MCI-ABneg = Aβ- MCI patients; Ctrl-ABpos = Aβ+ cognitively normal individuals, MCI- ABpos = Aβ+ MCI patients; AD = Dementia due to Alzheimer’s disease; q = false discovery rate adjusted p-value. Image generated using the ggplot package in R

**Fig. 2:**
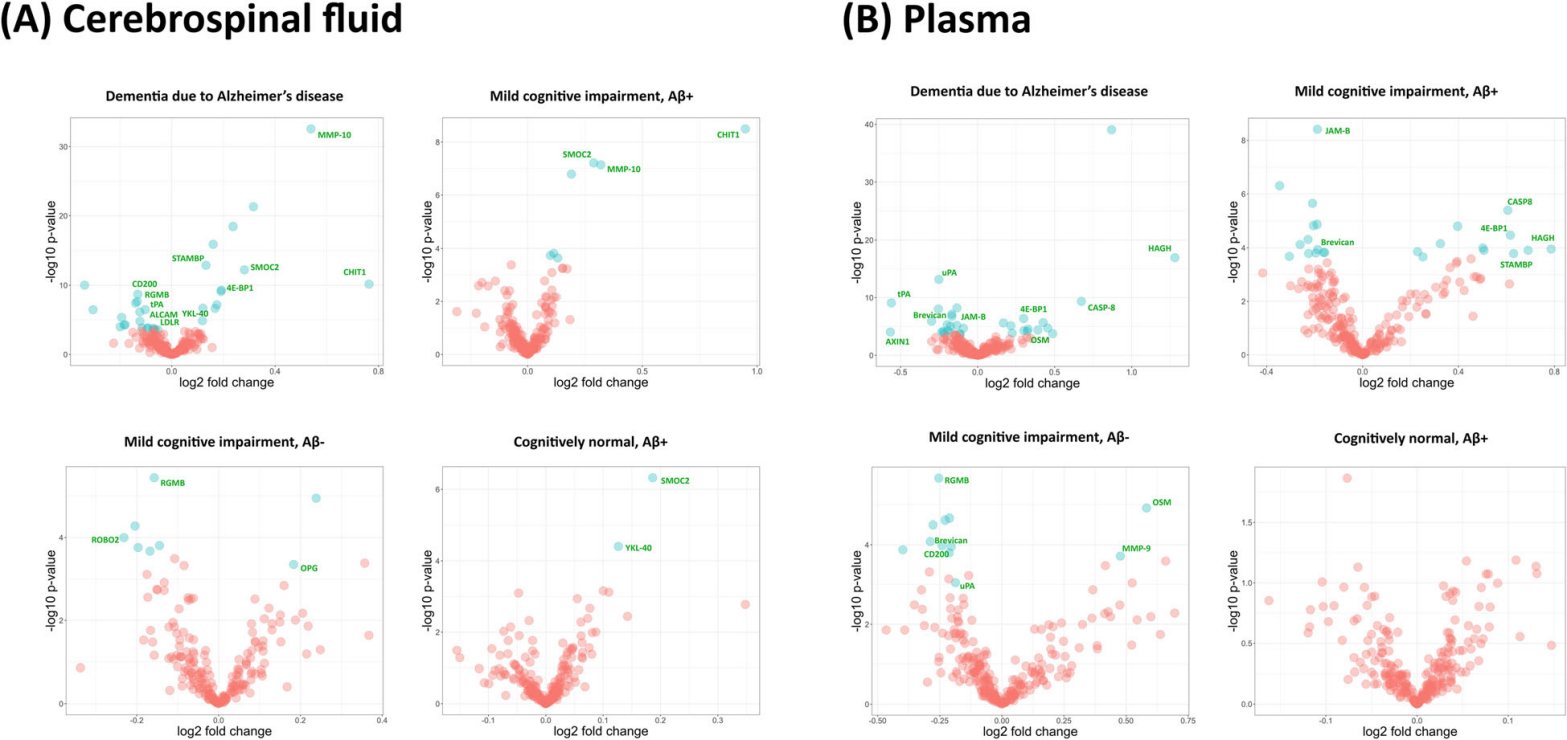
Volcano plots illustrating the log_2_-transformed fold change and -log_10_-transformed *p*-value (uncorrected) for all proteins assessed in **a** cerebrospinal fluid and **b** plasma. Proteins showing evidence of differential regulation after adjustment for false discovery rate (FDR) are denoted in blue. Proteins showing evidence of differential regulation in two or more patient groups, and/or showing replicated evidence of differential regulation in the Memory Lund replication sample, are noted in green text

Compared to Aβ- cognitively normal (CN) elderly controls, AD-dementia and Aβ+ MCI patients both showed significant increases of four proteins in CSF, including chitinase 1 (CHIT1; *d* = 0.95 in Aβ+ MCI; *d* = 0.76 in AD; *q* < 6.07 × 10^− 7^), matrix metalloproteinase 10 (MMP-10; *d* = 0.32 in Aβ+ MCI; *d* = 0.54 in AD; *q* < 1.16 × 10^− 5^), SMOC2 (*d* = 0.29 in Aβ+ MCI; *d* = 0.28 in AD; *q* < 1.16 × 10^− 5^), and ezrin (*d* = 0.13 in Aβ+ MCI; *d* = 0.24 *q* < 0.05; see Fig. 1a and Table 2). An additional five CSF proteins were increased, and 13 CSF proteins were decreased, in AD-dementia patients only, while an additional three proteins were increased in Aβ+ MCI patients only, when compared to controls (see Fig. 2a and Table 2).

**Table 2:**
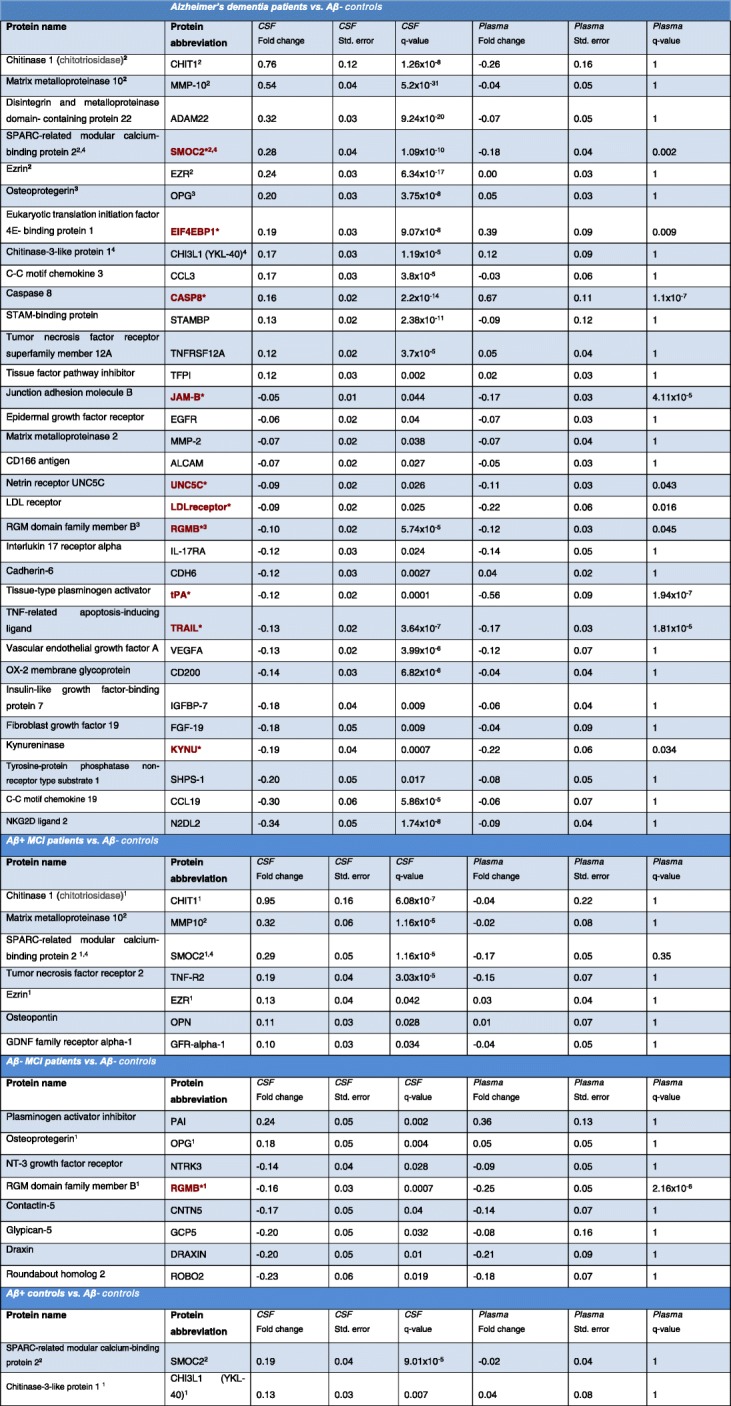
Demographic and clinical data for the Memory Malmö ‘discovery’ cohort

Red text with asterisk* = Protein is differentially regulated in both plasma and CSF

^1^Protein is also differentially regulated in AD-dementia patients

^2^Protein is also differentially regulated in AB+ MCI patients

^3^Protein is also differentially regulated in AB- MCI patients

^4^Protein is also differentially regulated in AB+ CN elderly individuals

### CSF levels of SMOC2 and YKL-40 were increased in Aβ-positive, cognitively normal individuals

Two proteins were increased in the CSF of Aβ+ cognitively normal (CN) individuals, including SMOC2 (*d* = 0.19; *q* = 9.01 × 10^− 5^), which was also increased in the CSF of Aβ+ MCI patients (*d* = 0.29; *q* = 1.16 × 10^− 5^) and AD-dementia patients (*d* = 0.28; *q* = 1.09 × 10^− 10^), and YKL-40 (*d* = 0.13; *q* = 0.007), which was also increased in the CSF of AD-dementia patients (*d* = 0.17; *q* = 1.19 × 10^− 5^; see Fig. 1a and Table 2).

### CSF levels of OPG were increased, and RGMB were decreased, in AD and Aβ-negative MCI

Compared to Aβ- CN, CSF concentrations of osteoprotegerin (OPG) were significantly increased in both Aβ- MCI patients (*d* = 0.23; *q* = 0.004) and patients with AD (*d* = 0.2; *q* = 3.75 × 10^− 8^; see Fig. 1a and Table 2). Aβ- MCI patients also showed significant decreases of repulsive guidance molecule B (RGMB; *d* = − 0.16; *q* = 0.0007), which was decreased in AD (*d* = − 0.1; *q* = 5.74 × 10^− 5^; see Fig. 1a and Table 2). An additional six proteins were differentially regulated in Aβ- MCI patients only (see Fig. 2a and Table 2).

### Findings were replicated for eleven proteins in CSF

Altered levels of 11 CSF proteins, as observed in our primary analysis, were replicated in an independent cohort of 179 individuals. In the replication sample, CHIT1 CSF levels were significantly up-regulated in Aβ+ MCI patients (*d* = 1.16, *p* = 4.9 × 10^− 6^, unadjusted), Aβ- MCI patients (*d* = 0.8, *p* = 0.003, unadjusted), and Aβ+ CN individuals (*d* = 1.28, *p* = 2.63 × 10^− 5^, unadjusted) compared to Aβ- CN. SMOC2 CSF levels were also increased in Aβ+ MCI patients (*d* = 0.4, *p* = 2 × 10^− 3^, unadjusted) and Aβ- + CN individuals (*d* = 0.45, *p* = 5 × 10^− 3^, unadjusted) compared to Aβ- CN. CSF levels of LDL receptor were modestly decreased in Aβ+ MCI patients (*d* = − 0.09, *p* = 0.049, unadjusted) and Aβ+ CN individuals (*d* = − 0.14, *p* = 0.007, unadjusted). CSF levels of CD200 were also modestly decreased in Aβ+ MCI patients *d* = − 0.13, *p* = 0.043, unadjusted) and Aβ- MCI patients (*d* = − 0.15, *p* = 0.03, unadjusted). Compared to Aβ- CN, Aβ+ MCI patients showed increased CSF levels of MMP-10 (*d* = 0.32, *p* = 0.008, unadjusted) and EIF4EBP1 (*d* = 0.13, *p* = 0.038, unadjusted), and decreased CSF levels of CD166 antigen (ALCAM; *d* = − 0.12, *p* = 0.005, unadjusted). Compared to controls, ROBO2 (*d* = − 0.27, *p* = 0.004, unadjusted) and RGMB protein (*d* = − 0.11, *p* = 0.049, unadjusted) were decreased in the CSF of Aβ- MCI patients, whereas tissue plasminogen activator (tPA) was decreased (*d* = − 0.2, *p* = 0.004, unadjusted) and STAM-binding protein (STAMBP) was increased (*d* = 0.09, *p* = 0.03, unadjusted) in Aβ+ MCI patients (see Fig. 2a and Additional file 1: Table S7).

### Plasma levels of HAGH, SIRT2, CASP8, EIF4EBP1, and IL-8 were increased in AD and Aβ-positive MCI

Compared to Aβ- CN, five plasma proteins were significantly increased in both AD and Aβ+ MCI patients, including HAGH (*d* = 0.79 in Aβ+ MCI; *d* = 1.28 in AD; *q* < 0.05), sirtuin-2 (SIRT2; *d* = 0.69 in Aβ+ MCI; *d* = 0.49 in AD; *q* < 0.05), caspase 8 (CASP8; *d* = 0.62 in Aβ+ MCI; *d* = 0.67 in AD; *q* < 0.01), EIF4EBP1 (*d* = 0.51 in Aβ+ MCI; *d* = 0.39 in AD; *q* < 0.05), and interleukin-8 (IL-8; *d* = 0.32 in Aβ+ MCI; *d* = 0.3 in AD; *q* < 0.05; see Fig. 1b and Table 3). AD-dementia and Aβ+ MCI patients showed additional increases in two distinct sets of six proteins, as detailed in Table 3.

**Table 3:**
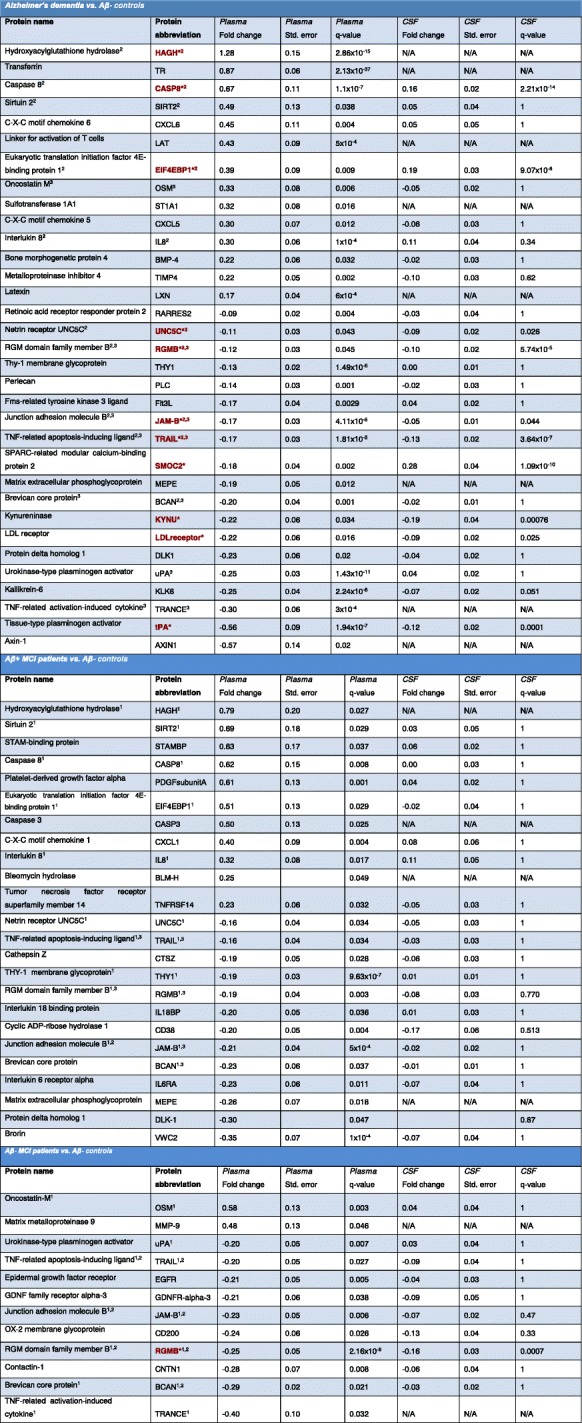
Differentially regulated proteins in human plasma

Red text with asterisk* = Protein is differentially regulated in both plasma and CSF

^1^Protein is also differentially regulated in AD-dementia patients

^2^Protein is also differentially regulated in AB+ MCI patients

^3^Protein is also differentially regulated in AB- MCI patients

### Plasma levels of JAM-B, brevican, THY-1, RGMB, UNC5C and TRAIL were decreased in AD and Aβ-positive MCI

AD and Aβ+ MCI patients showed decreases in six plasma proteins, including JAM-B (*d* = − 0.23 in Aβ+ MCI; *d* = − 0.21 in AD; *q* < 0.001), brevican (*d* = − 0.2 in Aβ+ MCI; *d* = − 0.23 in AD; *q* < 0.05), thy-1 membrane glycoprotein (THY1; *d* = −-0.19 in Aβ+ MCI; *d* = − 0.13 in AD; *q* < 1 × 10^− 5^), RGMB (*d* = − 0.19 in Aβ+ MCI; *d* = − 0.12 in AD; *q* < 0.05), UNC5C (*d* = − 0.16 in Aβ+ MCI; *d* = − 0.11 in AD; *q* < 0.05), and TRAIL (*d* = − 0.16 in Aβ+ MCI; *d* = − 0.17 in AD; *q* < 0.05); see Fig. 1b and Table 3. An additional 13 proteins were decreased in AD patients only, and seven proteins were decreased in Aβ+ MCI patients only, when compared to Aβ- controls (see Table 3).

The Aβ+ CN group did not show any significant differential regulation of proteins in plasma when compared to Aβ- CN (*q* > 0.05).

### Plasma levels of OSM were increased, and RGMB, JAM-B, TRAIL, TRANCE and brevican were decreased, in AD and Aβ-negative MCI

Compared to Aβ- CN, Aβ- MCI patients showed increased levels of oncostatin M (OSM; d = 0.58; q = 0.003) in plasma, which were also significantly increased in AD (*d* = 0.33; *q* = 0.006; see Table 3).

AD, Aβ+ MCI, and Aβ- MCI patients all showed decreases of three proteins in plasma, including RGMB (*d* = − 0.25 in Aβ- MCI; *d* = − 0.19 in Aβ+ MCI; *d* = − 0.12 in AD; *q* < 0.05), JAM-B (*d* = − 0.23 in Aβ- MCI; *d* = − 0.21 in Aβ+ MCI; *d* = − 0.17 in AD; *q* < 0.001), TRAIL (*d* = − 0.2 in Aβ- MCI; *d* = − 0.16 in Aβ+ MCI; *d* = − 0.17 in AD; *q* < 0.05).

Two proteins were decreased in both Aβ- MCI and AD compared to Aβ- controls, including TNF-related activation-induced cytokine (TRANCE; *d* = − 0.4 in Aβ- MCI; *d* = − 0.3 in AD; *q* < 0.05) and brevican (*d* = − 0.29 in Aβ- MCI; *d* = − 0.2 in AD; *q* < 0.05); see Fig. 2b and Table 3.

### Findings were replicated for six proteins in plasma

In an independent sample of 179 individuals, we observed differential regulation of seven proteins that were also differentially regulated in our primary analysis. Plasma levels of OSM were increased in Aβ+ MCI patients (*d* = 0.37, *p* = 0.035, unadjusted) and Aβ- MCI patients (*d* = 0.56, *p* = 0.002, unadjusted) compared to Aβ- CN. Plasma MMP-9 levels were increased in Aβ+ MCI patients (*d* = 0.41, *p* = 0.01, unadjusted) and Aβ- MCI patients (*d* = 0.55, *p* = 0.003, unadjusted) compared to Aβ- CN. Increased levels of HAGH (*d* = 0.6, *p* = 0.0061, unadjusted) and CD200 (*d* = 0.15, *p* = 0.02, unadjusted) were also observed in Aβ+ CN compared to Aβ- CN. Pronounced decreases of AXIN1 were observed in the plasma of Aβ+ MCI patients (*d* = 0.51, *p* = 0.02, unadjusted), Aβ- MCI patients (*d* = − 0.77, *p* = 0.002, unadjusted), and Aβ+ CN individuals (*d* = − 0.77, *p* = 0.005, unadjusted) compared to Aβ- CN. Finally, plasma levels of urokinase-type plasminogen activator (uPA) were decreased in Aβ+ MCI patients (*d* = − 0.14, *p* = 0.02, unadjusted), Aβ- MCI patients (*d* = − 0.16, *p* = 0.043, unadjusted), and Aβ+ CN individuals (*d* = − 0.14, *p* = 0.044, unadjusted) compared to Aβ- CN (see Fig. 2b and Additional file 1: Table S8).

### CSF levels of MMP-10 and YKL-40 negatively associated with baseline cortical thickness levels

Quality inspection identified four MCI patients and one control with segmentation errors; thus, 410 participants were included in the final MRI analysis.

AD-signature cortical thickness [17] was negatively associated with four proteins in CSF, including MMP-10 and YKL-40 (*q* < 0.05), both of which were differentially regulated in the cross-sectional analyses, and two proteins in plasma, including elafin and lymphotoxin-beta receptor (*q* < 0.01; Additional file 1: Table S9). Baseline hippocampal volume was not significantly associated with protein levels in plasma or CSF (*q* > 0.05).

### Multiple differentially regulated CSF and plasma proteins associated with cognitive performance

Baseline CDR-SB scores were significantly associated with levels of sixteen proteins in CSF, including SMOC2, IL8, MMP-10, CCL3, TNF-R2, ezrin, RGMB and OPN, all of which were differential regulated in our univariate analysis (*q* < 0.05; Additional file 1: Table S10). Baseline CDR-SB score was also associated with concentrations of eight proteins in plasma, including positive associations with IL-8 and TIMP4, and negative associations with JAM-B, THY1 and latexin, all of which were differentially regulated in our univariate analysis (*q* < 0.05; Additional file 1: Table S11). Baseline MMSE scores negatively associated with levels of four proteins in plasma, including IL8 and PDF subunit alpha (− 0.1 < *b* < − 0.05; *q* < 0.05), and positively associated with levels of fifteen proteins in plasma, including THY1, RGMB, brevican, brorin, kallikrein-6, bone morphogenetic protein 4, TRANCE, and delta homolog 1 (*q* < 0.05; Additional file 1: Tables S12-S13). In CSF, MMP-10, OPN and TNF-R2 were nominally associated with baseline MMSE score, but did not survive FDR adjustment (*q* > 0.05).

### Approximately half of CSF proteins correlated modestly with their analogs in plasma

**Fig. 3:**
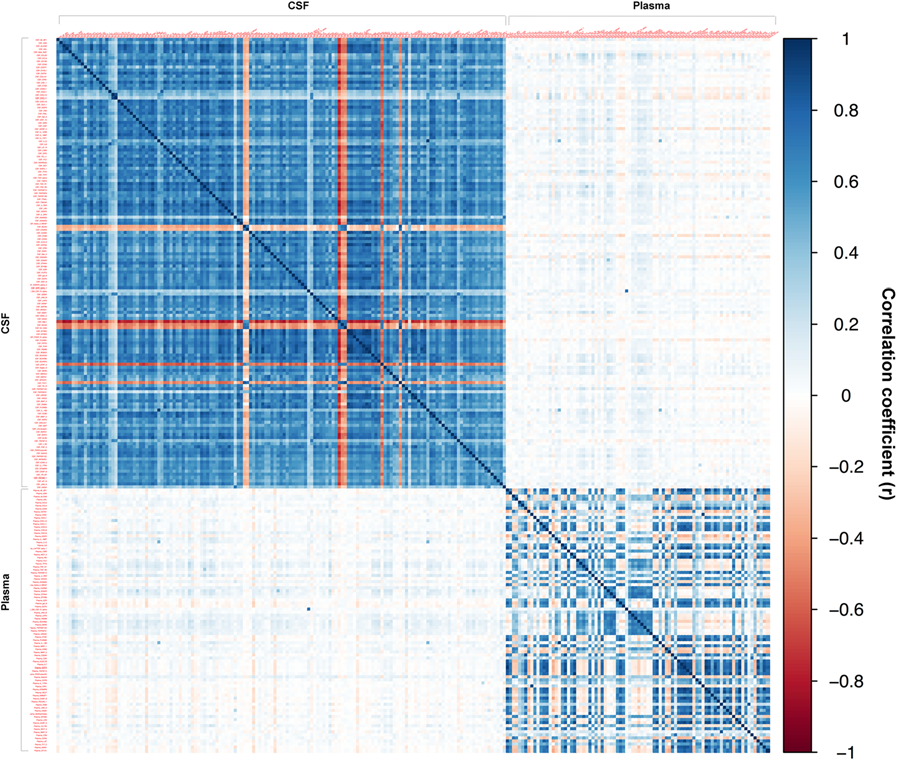
Between- and within-tissue correlation matrix, representing the strength of correlation (Pearson’s *r*) between proteins in plasma and cerebrospinal fluid (CSF). Positive correlations are illustrated in blue; negative correlations are illustrated in red. To reduce the dimensionality of the correlation matrix, only those proteins that were above the limit of detection, yielding Pearson’s r values equal to or greater than 0.7, were included. Image generated using the ggplot package in *R*

Overall, the strongest protein-protein correlations were observed within fluids, as illustrated in Fig. 3 and Additional file 1: Table S14. Between fluids, moderate-to-strong, positive correlations were observed for 14 proteins in CSF and their corresponding analogs in plasma, including CHIT1, which was significantly up-regulated in our primary univariate analysis of patients with AD-dementia and Aβ+ MCI versus Aβ- controls (0.5 < *r* < 0.73; *q* < 1 × 10^− 61^). Weak-to-modest positive correlations were observed across an additional 123 proteins in CSF and their analogs in plasma, including 35 proteins showing evidence of differential regulation in our univariate cross-sectional analyses (0.08 < *r* < 0.49; *q* < 0.05; Additional file 1: Table S14).

### Multivariate modeling predicted early AD with high accuracy in CSF

A series of multivariate LASSO regression models produced high predictive accuracy estimates in CSF for AD dementia, in a model that included 36 biomarkers (AUC = 0.95, 95%; CI = 0.9–0.99), and for Aβ+ MCI, in a model that included 43 biomarkers and gender (AUC = 0.92; 95% CI = 0.80–0.98). AUCs for CSF models were non-significant for Aβ- MCI (AUC = 0.78, 95% CI = 0.46–0.98), and low for Aβ+ CN, in a model that included 14 biomarkers and gender (AUC = 0.64, 95% CI = 0.57–0.7; Additional file 1: Table S2).

### Multivariate modeling predicted AD with high accuracy, and MCI with moderate accuracy, in plasma

In plasma, multivariate regression produced high AUCs for AD dementia, in a model that included 74 biomarkers and age (AUC = 0.94, 95% CI = 0.87–0.98). Predictive accuracy was moderate for Aβ+ MCI, in a model that included 53 biomarkers and age (AUC = 0.78, 95% CI = 0.68–0.87; see Additional file 1: Table S3). The plasma models were not significant for Aβ- MCI (AUC = 0.76, 95% CI = 0.47–0.92) or Aβ+ CN (AUC = 0.58, 95% CI = 0.47–0.72).

## Discussion

In one of the broadest multiplex proteomics studies of dementia conducted to date, we identified a series of significant alterations across plasma and CSF, implicating multiple proteins involved in regulation of the inflammatory response, apoptosis, endocytosis, leukocyte proliferation, and other biological processes believed to be downstream of, and potentially orthogonal to, Aβ and tau deposition. A subset of proteins collectively discriminated individuals with AD dementia from healthy controls with 95% predictive accuracy in CSF, and 94% accuracy in plasma, representing an improvement over prior multiplex panels [13, 16, 36, 42, 43]. With the exceptions of YKL-40 [1, 11, 18] and CHIT1 [27] in CSF, and MMP-9 [7] in blood, our findings highlight a set of relatively unexplored candidate biomarkers of neurodegeneration, warranting further validation in independent disease cohorts using targeted proteomic assays.

Our findings highlight the roles of several functionally pleiotropic proteins in AD, such as CASP8, which was increased in the plasma and CSF of AD-dementia patients, and in the plasma of Aβ+ MCI patients. CASP8 is involved in synaptic plasticity, amyloid processing, memory, and regulation of microglial pro-inflammatory activation [60]. The *CASP8* gene has shown a significant rare variant burden association with AD susceptibility [46], and prior immunohistochemical analyses have revealed activation of CASP8 in AD hippocampal tissue [47]. Caspase inhibition has been proposed as a mechanism to promote neuronal survival [49], and may be a beneficial therapeutic strategy for AD.

Similarly, JAM-B, a protein involved in synaptic adhesion, lymphocyte transendothelial migration [21], and vascular inflammation [59], was down-regulated in the plasma and CSF of AD patients and the plasma of MCI patients compared with cognitively normal Aβ- controls. In plasma, JAM-B levels negatively associated with baseline CDR-SB scores. Polymorphisms in or near the *JAM2* gene have shown suggestive associations with cognitive performance [34], postmortem Aβ load [51], and longitudinal changes in amyloid plaque burden on PET [44].

CSF concentrations of MMP-10 were elevated in Aβ+ MCI and AD patients, positively correlated with cognitive performance, and negatively associated with AD-signature cortical thickness. Other matrix metalloproteinase-related proteins, including MMP-9, TIMP-4, and ADAM22, were also up-regulated in AD. These findings highlight the complex pathophysiological role of endopeptidases in AD, potentially via direct degradation of Aβ deposits [32] or indirect mediation of Aβ-induced blood-CSF barrier dysfunction [6]. Transferrin receptor, which facilitates brain iron uptake, was also significantly increased in the plasma of AD patients, implicating elevated brain iron levels, potentially via blood-brain barrier (BBB) breakdown, in disease pathophysiology [54]. Further investigation of the role of metalloproteinases, endothelial proteins and adhesion molecules, particularly as they relate to meningeal lymphatics, BBB function and iron levels in AD [54], may facilitate novel therapeutic target discovery.

In the CSF and plasma of AD patients, we found decreased concentrations of tPA, a blood clotting enzyme which co-localizes with amyloid-rich regions and phosphorylated tau in post-mortem AD brains [30], and has been linked to accelerated Aβ degradation in-vivo [37]. In AD plasma, we found decreased levels of another plasminogen activator, uPA, which has been associated with inhibition of Aβ neurotoxicity and fibrillogenesis [56] and recovery after axonal injury [31].

Other notable proteins that showed evidence of differential regulation in AD compared to healthy elderly individuals included CD200, a glycoprotein previously associated with enhanced in-vivo and in-vitro amyloid phagocytosis [57], UNC5, an axonal guidance protein receptor which has previously shown genetic links to familial, late-onset AD via increased susceptibility to neuronal cell death [61], and SMOC2, a member of the SPARC protein family, which is involved in microgliosis and functional recovery after cortical ischemia [24], and was significantly associated with cognitive performance in our study. We also observed profound increases of HAGH, also known as glyoxalase II, in plasma, reemphasizing the potential role of advanced glycation end products in neurodegeneration [23, 63].

Our study highlights the changes and potential contributions of several chemokines (CXCL1, CCL3, CXCL5, CXCL6, CCL3, CCL19), interleukins (IL8, IL6-RA, IL18-BP), and other immune markers (OSM, ALCAM, OPG, CHIT1, YKL-40, STAMBP, MMP-9, MMP-10) in the prodromal and dementia phases of AD. YKL-40, a chitinase expressed by astrocytes and microglia in the CNS [8], was increased in the CSF of Aβ+ CN individuals and patients with AD, and negatively associated with thickness measures in AD-vulnerable brain regions, replicating prior findings [2] and reemphasizing similar findings from the same cohort, using a standard ELISA assay [19]. However, elevations of YKL-40 in CSF were modest compared with prior investigations [1, 11, 18] and were negligible in plasma, indicating limited diagnostic utility of this biomarker in isolation [27]. CHIT1, another putative marker of microglial activation, showed more pronounced elevations in the CSF of Aβ+ MCI and AD patients, supporting prior observations of up-regulated CHIT1 in progressive brain illnesses [53, 58], and warranting further investigation of the role of chitinases in immune response and neurodegeneration.

Compared with Aβ- CNs, MCI patients without evidence of pathological CSF Aβ showed significant differential regulation of OPG, RGMB and ROBO2 protein in CSF, and differential regulation of multiple proteins in plasma, including brevican. OPG, RGMB, and ROBO2 have been implicated in CNS development, axonal growth, and recovery after nerve injury [15, 25, 50], whereas upregulation of brevican has been observed in the CNS during the consolidation of long-term memories [35]. Thus, dysregulation of these proteins in Aβ- MCI may reflect broader mechanisms involved in neurodegeneration, potentially unrelated to Aβ pathology.

Conversely, certain proteins, dysregulated when Aβ+ patients were compared to Aβ- controls, may reflect the presence of Aβ deposits rather than a specific diagnosis of AD or MCI. To address this possibility, we conducted a series of supplementary analyses, as detailed in Additional file 1: Note 1. These analyses revealed that, with the exceptions of CHIT1, YKL-40 and SMOC2 in CSF, most differentially regulated proteins identified in Aβ+ patients may represent markers of disease diagnosis, rather than Aβ deposition alone, as they showed similar patterns of differential regulation when Aβ+ groups were compared with one another directly (see Additional file 1: Tables S23-S32).

Some differentially regulated proteins may reflect the influences of coexisting pathologies in AD, such as Lewy body inclusions or TDP-43 aggregates. To test this hypothesis, we conducted supplementary analyses in a small, independent cohort of Aβ- healthy elderly individuals compared with Aβ- patients with Parkinson’s disease (PD), multiple system atrophy (MSA), and progressive supranuclear palsy (PSP; see Additional file 1: Note 1). This analysis indicated that MMP-10, STAMBP, LDL receptor, and EIF4EBP1 likely represent disease-specific biomarkers of AD, whereas CHIT1, ALCAM, CD200, OPG, ROBO2, and RGMB may serve as broader neurodegeneration biomarkers, potentially influenced by coexisting pathologies beyond Aβ- and tau (Additional file 1: Tables S33-S38). Further studies of patients with Lewy body dementia, frontotemporal dementia, and other neurodegenerative illnesses are strongly recommended to help validate these findings.

Despite its scale, our study has limitations. Many differentially regulated proteins yielded small-to-modest effect sizes (0.05 < *d* < 0.4), and the functions of some proteins demonstrating AD-related changes are not fully understood. Also, given the study’s cross-sectional design, we could not investigate the precise temporal dynamics of important biological processes such as innate immunity in AD. Future investigations will combine longitudinal plasma and CSF measurements with genome-wide genotyping to disentangle causal from consequential biomarkers, and to map the development of protein biomarkers throughout the progression of disease. Additionally, the Olink™ PEA technology takes advantage of unique tags and polymerase chain reaction to measure up to 92 proteins in parallel, including many low-expressing analytes typically undetected using traditional multiplex assays. The technology therefore represents a relatively focused screen, whereby protein levels are reported as normalized protein concentration (NPX). Prospective experiments should combine more unbiased proteomics approaches (such as mass spectrometry) with targeted assays (such as enzyme-linked immunoabsorbance assays, single molecule arrays, or customized Olink™ panels), reporting results as absolute concentrations.

Finally, while we identified subsets of proteins that accurately discriminated MCI and AD patients from elderly controls, further work is required to identify a suitable selection of biomarkers for very early detection of cognitive impairment, prior to the presentation of overt clinical symptoms. Replication studies in independent cohorts, complemented by larger multiplex proteomics panels, genetic risk scores, advanced neuroimaging, neuropsychological assessments, and post-mortem expression studies will help characterize early contributors towards longitudinal cognitive decline in AD [33].

## Conclusion

In conclusion, we have implicated multiple markers of neuroinflammation, cerebrovascular dysfunction, apoptosis, and other CNS processes in the preclinical, prodromal, and dementia phases of AD. Approximately one third of proteins successfully assayed in CSF significantly correlated with their analogs in plasma, and several differentially regulated proteins were associated with cortical atrophy and cognitive performance. These findings inform our understanding of AD biology, and indicate that blood-based biomarker panels may facilitate AD diagnosis, pending further refinement and validation.

## Additional file

Additional file 1: Supplementary Materials, Part 1: Notes S1-S2, Figures S1-S8, and Tables S1-S6. Supplementary Materials, Part 2: Tables S7-S39. (ZIP 789 kb)

## Data Availability

Anonymized data will be shared by request from a qualified academic investigator for the sole purpose of replicating procedures and results presented in the article and as long as data transfer is in agreement with EU legislation on the general data protection regulation and decisions by the Ethical Review Board of Sweden and Region Skåne.
