# Supplementary material for: Multiplex proteomics identifies novel CSF and plasma biomarkers of early Alzheimer’s disease
Source: Acta Neuropathol Commun. 2019 Nov 6;7:169. doi: 10.1186/s40478-019-0795-2 (PMC6836495; doi:10.1186/s40478-019-0795-2)
Supplement: Supplementary file 1 — Supplementary Materials, Part 1: Notes S1-S2, Figures S1-S8, and Tables S1-S6. Supplementary Materials, Part 2: Tables S7-S39. (ZIP 789 kb) [file 40478_2019_795_MOESM1_ESM.zip › Biofinder_Supplementary_Materials_ACTA_R1.docx]

Supplementary Information

**Multiplex proteomics identifies novel CSF and plasma biomarkers of early Alzheimer’s disease**

**Supplementary Note 1**. Supplementary statistical analyses

**Supplementary Note 2**. Multivariate LASSO regression: Adjusting for the standard covariates

**Supplementary Figure 1**. Principal component analysis plot for OLINK proteins in CSF (unjadusted)

**Supplementary Figure 2**. Principal component analysis plot for OLINK proteins in CSF (adjusting for age, sex, and mean protein concentration per subject)

**Supplementary Figure 3**. Principal component analysis plot for OLINK proteins in plasma (unjadusted)

**Supplementary Figure 4**. Principal component analysis plot for OLINK proteins in plasma (adjusting for age, sex, and mean protein concentration per subject)

**Supplementary Figure 5**. Scree plot showing the fraction of variance explained by each principal component in CSF (unadjusted)

**Supplementary Figure 6**. Scree plot showing the fraction of variance explained by each principal component in CSF (adjusting for age, sex, and mean protein concentration per subject)

**Supplementary Figure 7**. Scree plot showing the fraction of variance explained by each principal component in plasma (unadjusted)

**Supplementary Figure 8**. Scree plot showing the fraction of variance explained by each principal component in plasma (adjusting for age, sex, and mean protein concentration per subject)

**Supplementary Table S1**. Quality assessment for 270 proteins assayed across the OLINK Inflammation I, Cardiovascular III, and Neurology arrays

**Supplementary Table S2**. Cerebrospinal fluid proteins selected in multivariate LASSO analyses (main analysis, without adjusting for the standard covariates)

**Supplementary Table S3**. Plasma proteins selected in multivariate LASSO analyses (main analysis, without adjusting for the standard covariates)

**Supplementary Table S4**. Cerebrospinal fluid proteins selected in multivariate LASSO analyses (supplementary analysis, adjusting for the standard covariates)

**Supplementary Table S5**. Plasma proteins selected in multivariate LASSO analyses (supplementary analysis, adjusting for the standard covariates)

**Supplementary Table S6**. Demographic and clinical data for the Memory Lund ‘replication’ cohort.

**Supplementary Table S7**.* Cross-sectional CSF results from the Memory Lund ‘replication’ cohort (unadjusted p-values).

**Supplementary Table S8**.* Cross-sectional plasma results from the Memory Lund ‘replication’ cohort (unadjusted p-values).

**Supplementary Table S9**.* Associations between protein biomarkers and neuroimaging endpoints

**Supplementary Table S10**.* Associations between CSF proteins and baseline CDR-SB scores

**Supplementary Table S11**.* Associations between plasma proteins and baseline CDR-SB scores

**Supplementary Table S12**.* Associations between CSF proteins and baseline MMSE scores

**Supplementary Table S13**.* Associations between plasma proteins and baseline MMSE scores

**Supplementary Table S14**.* Correlations between plasma proteins and their analogs in cerebrospinal fluid

**Supplementary Table S15**.* Full cross-sectional CSF results from the Memory Malmö ‘discovery’ cohort (AD vs. control)

**Supplementary Table S16**.* Full cross-sectional CSF results from the Memory Malmö ‘discovery’ cohort (AB+ MCI vs. control)

**Supplementary Table S17**.* Full cross-sectional CSF results from the Memory Malmö ‘discovery’ cohort (AB- MCI vs. control)

**Supplementary Table S18**.* Full cross-sectional CSF results from the Memory Malmö ‘discovery’ cohort (AB+ CN vs. control)

**Supplementary Table S19**.* Full cross-sectional plasma results from the Memory Malmö ‘discovery’ cohort (AD vs. control)

**Supplementary Table S20**.* Full cross-sectional plasma results from the Memory Malmö ‘discovery’ cohort (AB+ MCI vs. control)

**Supplementary Table S21**.* Full cross-sectional plasma results from the Memory Malmö ‘discovery’ cohort (AB- MCI vs. control)

**Supplementary Table S22**.* Full cross-sectional plasma results from the Memory Malmö ‘discovery’ cohort (AB+ CN vs. control)

**Supplementary Table S23**.* Supplementary analysis: AD versus amyloid-positive MCI (CSF)

**Supplementary Table S24**.* Supplementary analysis: AD versus amyloid-positive MCI (plasma)

**Supplementary Table S25**.* Supplementary analysis: Amyloid-positive MCI versus amyloid-positive cognitively normal (CSF)

**Supplementary Table S26**.* Supplementary analysis: Amyloid-positive MCI versus amyloid-positive cognitively normal (plasma)

**Supplementary Table S27**.* Supplementary analysis: AD versus amyloid-positive cognitively normal (CSF)

**Supplementary Table S28**.* Supplementary analysis: AD versus amyloid-positive cognitively normal (plasma)

**Supplementary Table S29**.* Supplementary analysis: Amyloid-positive versus amyloid-negative non-demented individuals (CSF, Malmö)

**Supplementary Table S30**.* Supplementary analysis: Amyloid-positive versus amyloid-negative non-demented individuals (plasma, Malmö)

**Supplementary Table S31**.* Supplementary analysis: Amyloid-positive versus amyloid-negative non-demented individuals (CSF, Lund)

**Supplementary Table S32**.* Supplementary analysis: Amyloid-positive versus amyloid-negative non-demented individuals (plasma, Lund)

**Supplementary Table S33**.* Supplementary analysis: Parkinson’s disease versus control – all amyloid-negative (CSF)

**Supplementary Table S34**.* Supplementary analysis: Parkinson’s disease versus control – all amyloid-negative (plasma)

**Supplementary Table S35**.* Supplementary analysis: Multiple system atrophy versus control – all amyloid-negative (CSF)

**Supplementary Table S36**.* Supplementary analysis: Multiple system atrophy versus control – all amyloid-negative (plasma)

**Supplementary Table S37**.* Supplementary analysis: Progressive supranuclear palsy versus control – all amyloid-negative (CSF)

**Supplementary Table S38**.* Supplementary analysis: Progressive supranuclear palsy versus control – all amyloid-negative (plasma)

**Supplementary Table S39**.* Participant demographics for the Neurology Lund cohort (PD, MSA, PSP and healthy elderly)

**Supplementary Tables S7-S21 are included in a separate Excel spreadsheet, titled ‘BIofinder_Supplementary_Tables_6-20.xls’.*

**Supplementary Note 1.** Supplementary statistical analyses

Rationale:

In the main text, we focused on comparing amyloid-negative healthy elderly controls to three clinical subgroups reflecting different stages of the disease process (CN-AB+ = preclinical AD, MCI-AB+ = prodromal AD, AD = dementia) and a fourth subgroup likely reflecting non-amyloid-related processes (MCI-AB-). Certain candidate biomarkers identified in this analysis may represent surrogate markers of amyloid-beta deposition, rather than a diagnosis of AD. Other candidate biomarkers may reflect the influences of coexisting pathologies, such as Lewy body inclusions or TDP-43 aggregates. To address these possibilities, we conducted a series of supplementary analyses, which included:

1. Supplementary Study 1: Comparisons of protein concentrations between the three amyloid-positive clinical groups, i.e. AD-AB+ versus MCI-AB+, AD-AB+ versus CN-AB+, and MCI-AB+ versus CN-AB+,
2. Supplementary Study 2: Comparisons of protein concentrations between all non-demented amyloid positive patients (MCI-AB+ and CN-AB+) versus all amyloid negative patients (MCI-AB- and CN-AB-), and
3. Supplementary Study 3: Comparisons between amyloid-negative healthy elderly individuals and (i) amyloid-negative PD patients, (ii) amyloid-negative multiple system atrophy (MSA) patients, and (iii) amyloid-negative progressive supranuclear palsy (PSP) patients.

Methods

Supplementary Studies 1 and 2 comprised the same participants from our main study. In Supplementary Study 3, the healthy elderly cohort consisted of 34 amyloid negative patients (20 female; mean age: 64.44 years, SD= 8.56 years), the PD cohort consisted of 119 amyloid negative patients (41 female; mean age: 64.39 years, SD= 9.91 years), the MSA cohort consisted of 25 amyloid-negative patients (13 female; mean age: 63.08 years, SD= 8.41 years), and the PSP cohort consisted of 15 amyloid-negative patients (9 female; mean age: 69.13 years, SD= 4.58 years; see **Supplementary Table S39**). All participants were recruited from the Neurology Lund clinic in Northern Sweden (part of the BioFINDER study); **Supplementary Table S39** provides full participant demographics; further information on patient recruitment and diagnoses is available at [www.biofinder.se](http://www.biofinder.se).

Patients with PD, MSA and PSP were selected from the Neurology Lund clinic because the number of individuals with non-amyloid-related conditions such as dementia with Lewy bodies (N=7) or frontotemporal dementia (N=4) was small in Memory Malmö, whereas Memory Lund consisted exclusively of MCI patients and individuals with subjective cognitive impairment.

In Supplementary Studies 1 and 2, we performed multiple logistic regressions, adjusting for the standard covariates as described in the Methods section of the main text. In Supplementary Study 3, we conducted multiple logistic regressions, adjusting for age, gender and cross-subject mean protein concentration, only. Information on medications and smoking status was not collected at the Neurology Lund clinic, and therefore could not be included in our comparisons of PD, MSA and PSP patients versus controls.

Results

Supplementary Study 1 revealed that 34 CSF proteins differentiated AD patients from AB+ cognitively normal elderly individuals; many proteins from the original comparison of AD patients with AB- cognitively normal individuals were re-identified, with the notable exceptions of CHIT1, YKL-40, SMOC2, LDL receptor, and tPA. Similarly, in plasma, fifteen proteins were differentially regulated in AD patients versus AB+ CNs, including many proteins from the original AD vs. AB- CN analysis, with the exceptions of OSM and AXIN1. Five CSF proteins differentiated MCI AB+ and AD dementia; four of these proteins also differentiated AD dementia from healthy elderly controls in our original analysis (ADAM22, 4EBP1, CASP8, TNFRS12A). In plasma, fourteen plasma proteins differentiated MCI AB+ and AD dementia, which included five proteins that also differentiated AD dementia from healthy elderly controls in our original analysis (transferrin, AXIN1, BMP-4, STAMPB, tPA; see Supplementary Table S24); q<0.05. Finally, two proteins differentiated MCI-AB+ from CN-AB+, including MMP-10 and TNF-R2, which showed evidence of differential regulation in our main analysis, and 25 proteins differentiated these groups in plasma, including multiple proteins that showed evidence of differential regulation in the main comparison of MCI-AB+ vs. CN-AB-; q<0.05.

Supplementary Study 2 revealed significant differential regulation of eight CSF proteins when all non-demented amyloid positive patients (MCI-AB+, CN-AB+) were compared to all non-demented amyloid negative patients (MCI-AB-, CN-AB-); q<0.05. Notably, this included CHIT1, SMOC2 and YKL-40, which were not differentially regulated in Supplementary Study 1, but were differentially regulated in our main analyses.

Supplementary Study 3 identified significant down-regulation of beta NGF and EPHB6 in PD CSF (q<0.05), neither of which were changed in AD or MCI patients versus controls. Twenty-eight proteins were significantly differentially regulated in the CSF of MSA patients versus elderly controls, including twelve proteins from our primary analysis of AD and MCI patients versus healthy controls: VEGFA, FGF-19, NTRK3, **RGMB**, UNC5C, CCL3, **CHIT1**, TNF-R2, **ALCAM**, CNTN5, ROBO2, and **OPG** (q<0.05). Twenty proteins were significantly differentially regulated in PSP patients versus elderly controls, including 11 proteins from our primary analysis of AD and MCI patients versus healthy controls: **OPG**, NTRK3, **ROBO2**, **RGMB**, **CD200**, SHPS-1, GCP5, UNC5C, **ALCAM**, and CNTN5 (p<0.05). Comparisons with plasma samples from healthy elderly individuals revealed significant differential regulation of MMP-10 in PD plasma, GFR alpha 1, ITGB2 and ezrin in MSA plasma, and ITGB2 in PSP plasma – none of which showed evidence of differential regulation in our primary analysis of AD and MCI patients versus elderly controls.

Conclusions

Findings from Supplementary Study 2 suggest that CSF levels of CHIT1, SMOC2 and YKL-40 may represent surrogate markers of AB deposition, rather than of AD diagnosis. Findings from Supplementary Study 1 indicate that the majority of other differentially regulated proteins in our main analysis most likely reflect AD diagnosis and not AB deposition alone. Findings from Supplementary Study 3 indicate that MMP-10, STAMBP, LDL receptor, and 4E-BP1 may represent disease-specific biomarkers of AD, as they remain unchanged in PD, MSA, or PSP versus controls. However, other proteins may represent broader biomarkers of neurodegeneration, as they showed evidence of differential regulation when healthy elderly individuals were compared to patients with MSA (CHIT1, RGMB, ALCAM, OPG) and PSP (OPG, ROBO2, RGMB, CD200, ALCAM). Results from Supplementary Study 3 should be interpreted with caution, as we could not adjust for medications or smoking status as per our primary analyses of AD and MCI patients. Thus, larger studies in more heterogeneous collections of patients with neurodegenerative illnesses are strongly recommended to help validation our supplementary findings.

**Supplementary Note 2.** Multivariate LASSO regression: Adjusting for the standard covariates

In this study, we conducted two different LASSO analyses to identify subsets of proteins that could accurately discriminate AD, MCI-AB+, MCI-AB-, and AB+ CN from AB- healthy elderly controls. To reduce the complexity of our data, the first LASSO analysis, as reported in the main text, adjusted for age and gender only. The second, supplementary LASSO analysis included the standard covariates – age, gender, medication use at baseline, subject-level mean OLINK protein concentration, and smoking status.

In our supplementary analysis including the standard covariates, AUCs for the CSF models were high for AD dementia (AUC=0.95, 95% CI=0.90-0.99) and Aβ+ MCI (AUC=0.89, 95% CI=0.81-0.95), and lower for Aβ- MCI (AUC=0.72, 95% CI=0.56-0.91) and Aβ+ CN (AUC=0.64, 95% CI=0.55-0.71). The AUCs for the plasma models were high for AD dementia (AUC=0.93, 95% CI=0.86-0.97), lower for Aβ+ MCI (AUC=0.75, 95% CI=0.56-0.92) and Aβ- MCI (AUC=0.79, 95% CI=0.63-0.98), and not significant for Aβ+ CN (AUC=0.56, 95% CI=0.46-0.62).

We note that the area under the receiver operator curve (AUC) estimates are comparable between the first analysis (Supplementary Tables S2-3) and the second analysis (reported here, and in Supplementary Tables S4-5), hence, we report the first analysis in the main text, as it captures a greater number of proteins.


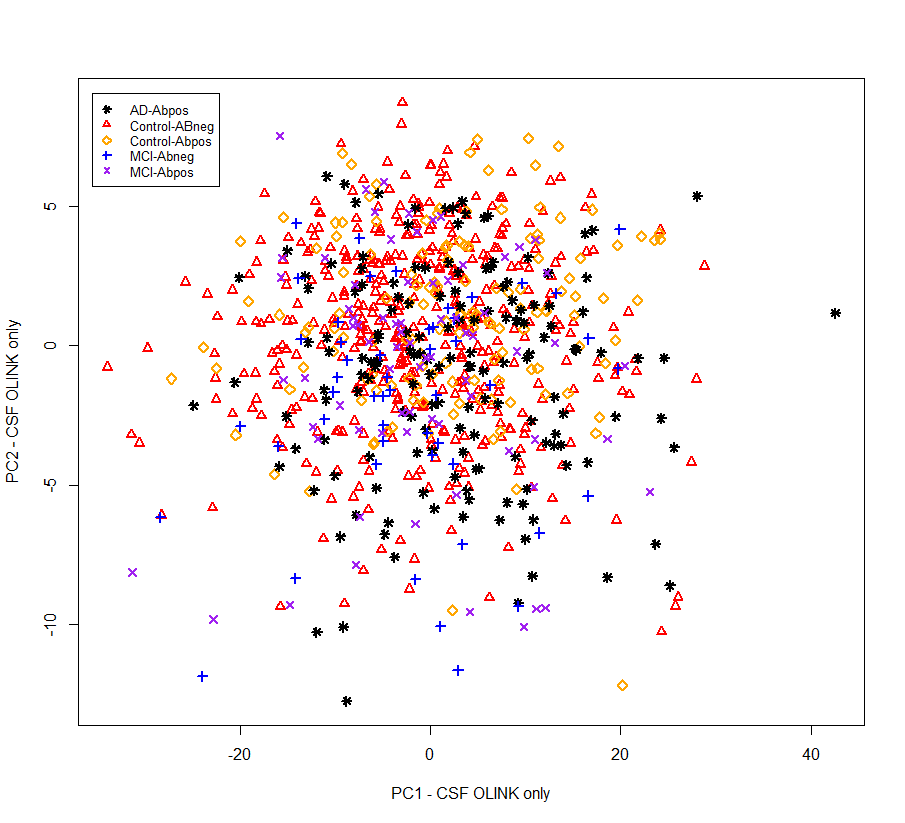


**Supplementary Figure 1**. Principal component analysis plot for OLINK proteins in CSF (unjadusted)

**
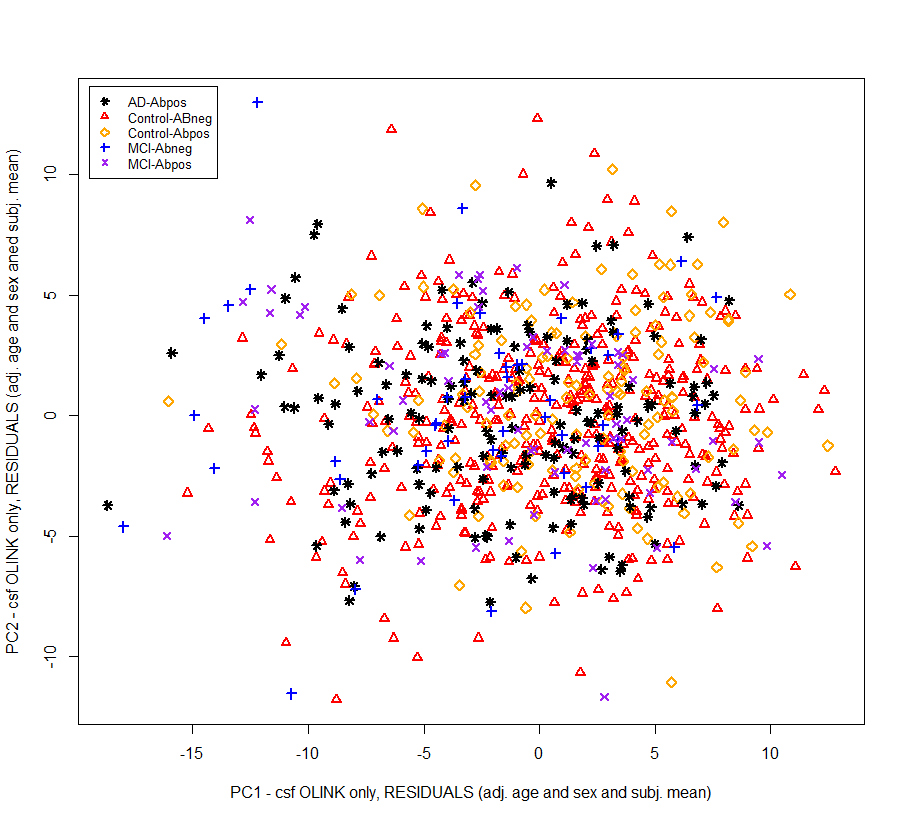
**

**Supplementary Figure 2**. Principal component analysis plot for OLINK proteins in CSF (adjusting for age, sex, and mean protein concentration per subject)


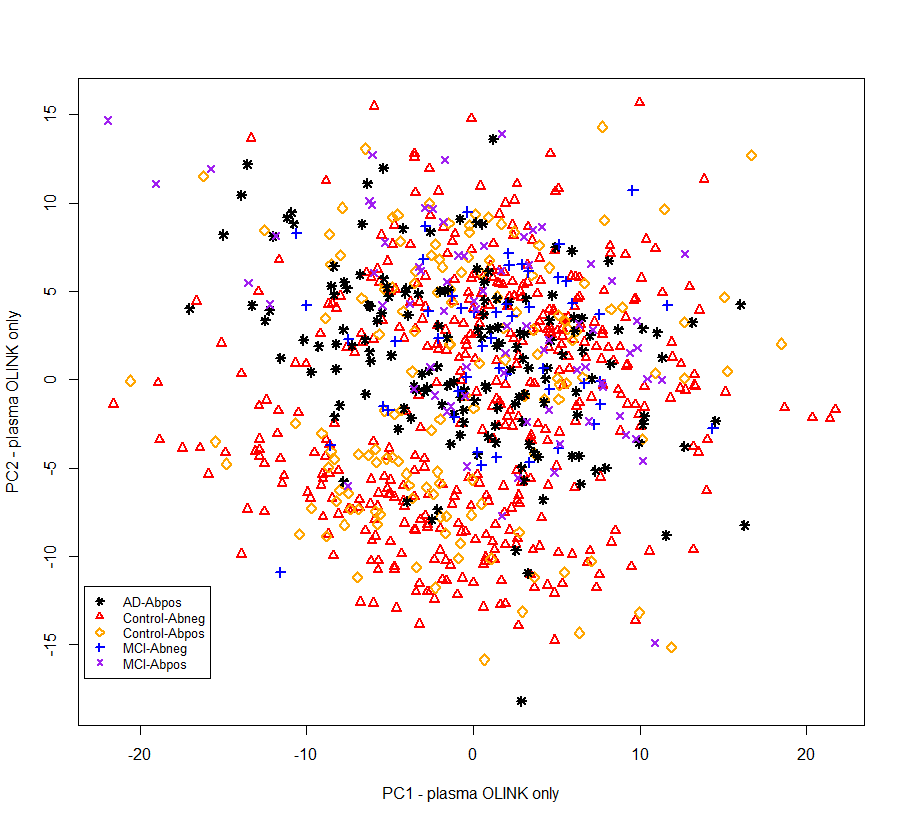


**Supplementary Figure 3**. Principal component analysis plot for OLINK proteins in plasma (unjadusted)

**
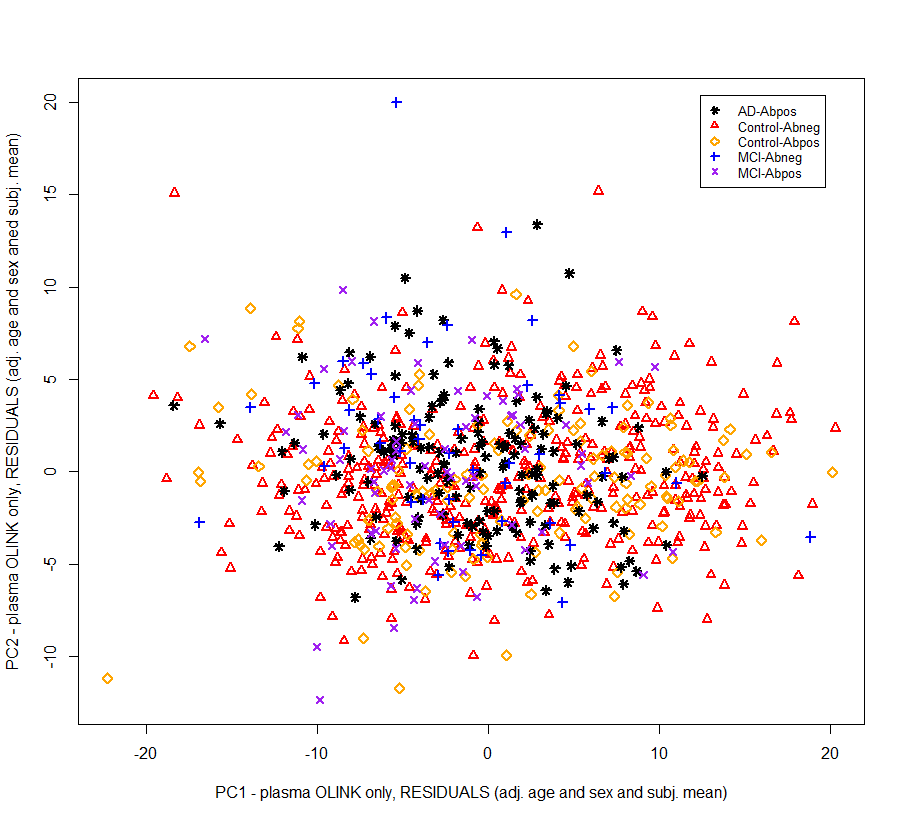
**

**Supplementary Figure 4**. Principal component analysis plot for OLINK proteins in plasma (adjusting for age, sex, and mean protein concentration per subject)

**
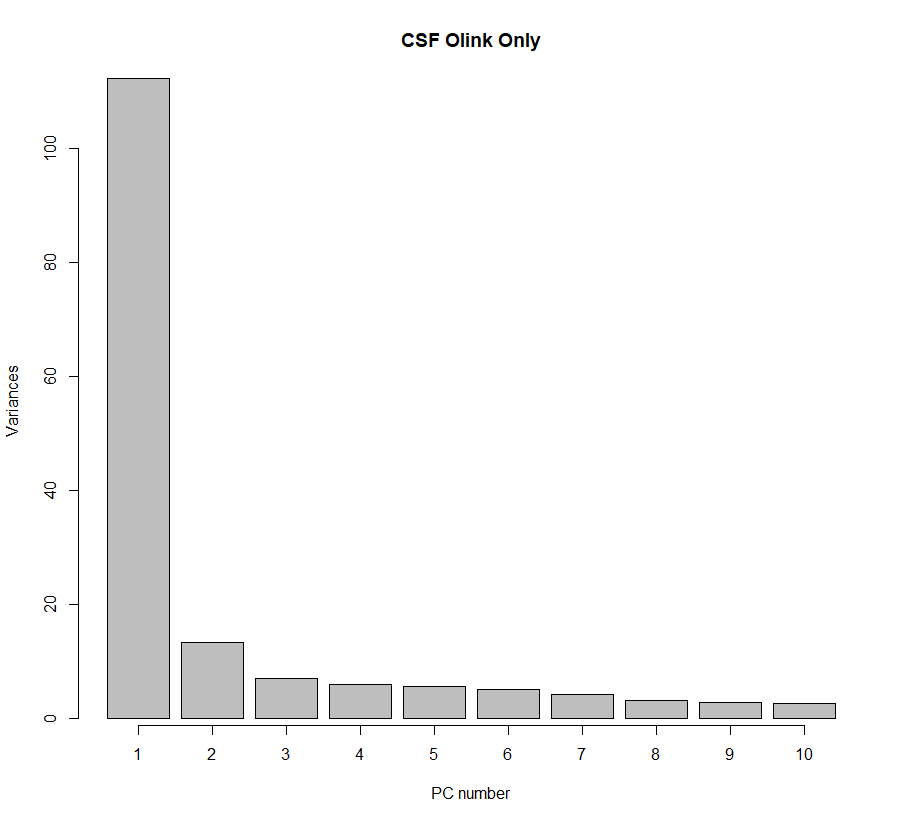
**

**Supplementary Figure 5**. Scree plot showing the fraction of variance explained by each principal component in CSF (unadjusted)


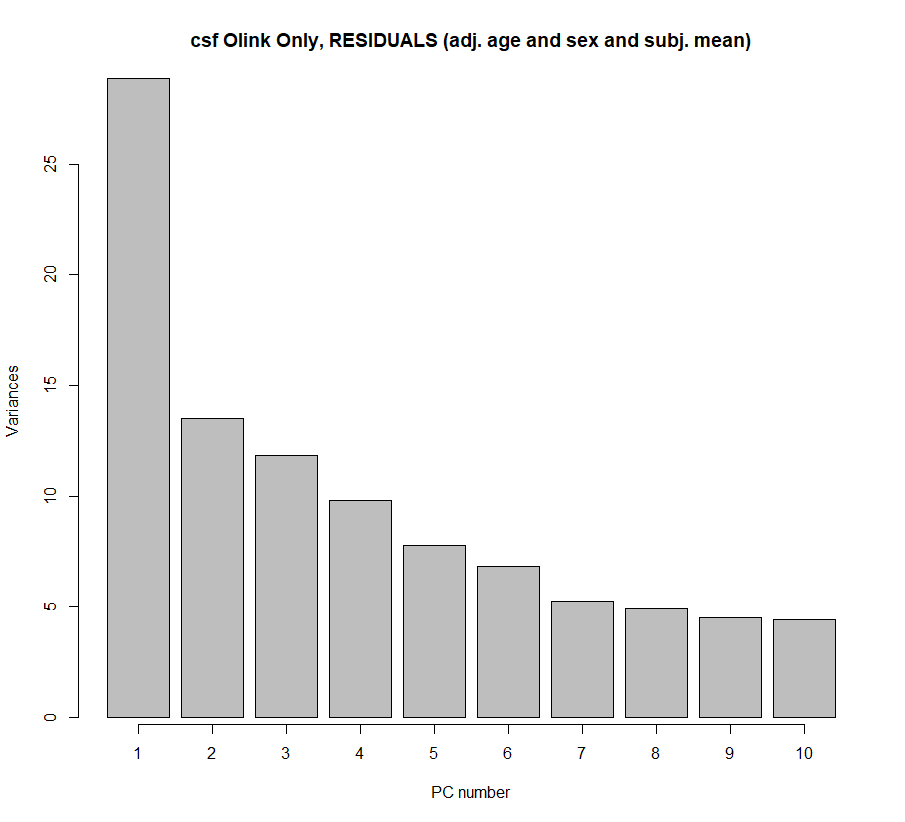


**Supplementary Figure 6**. Scree plot showing the fraction of variance explained by each principal component in CSF (adjusting for age, sex, and mean protein concentration per subject)

**
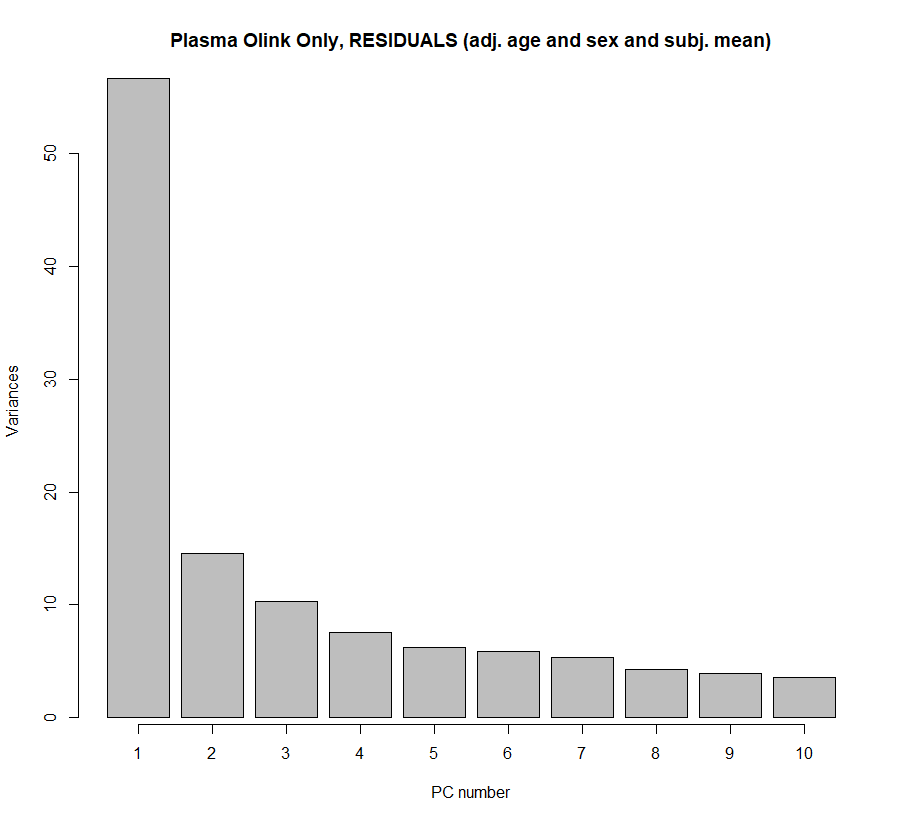
**

**Supplementary Figure 7**. Scree plot showing the fraction of variance explained by each principal component in plasma (unadjusted)

**
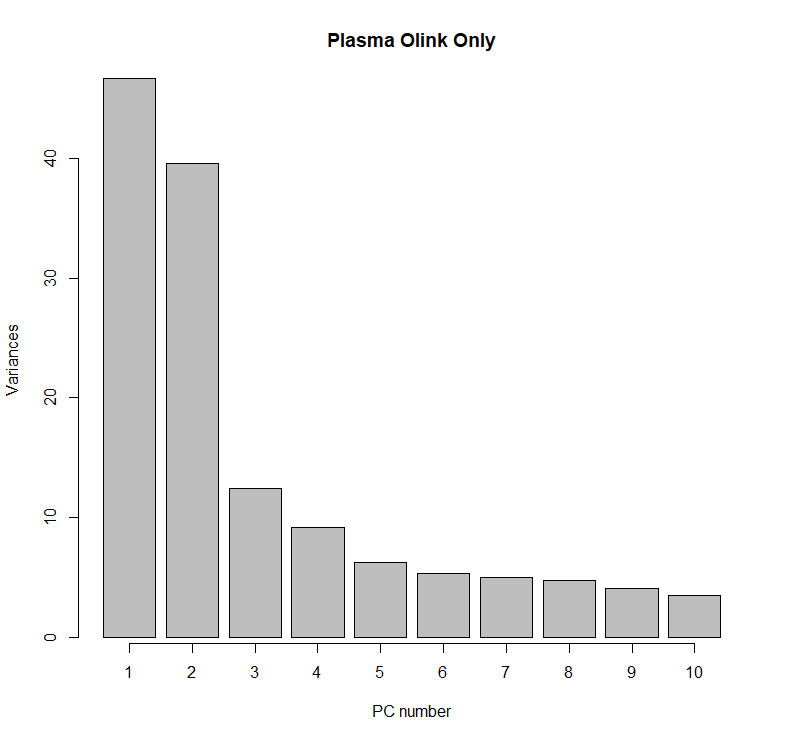
**

**Supplementary Figure 8**. Scree plot showing the fraction of variance explained by each principal component in plasma (adjusting for age, sex, and mean protein concentration per subject)

**Supplementary Table S1.** Quality assessment for 270 proteins assayed across the OLINK Inflammation I (INF I), Cardiovascular III (CVDIII), and Neurology I arrays (NEU I)

| Protein name | Abbreviation | Panel(s) | Detection rate (CSF; %) | QC procedure (CSF) | Detection rate (plasma; %) | QC procedure (plasma) |
| --- | --- | --- | --- | --- | --- | --- |
| Eukaryotic translation initiation factor 4E- binding protein 1 | EIF4EBP1 | INF I | 100.00 | Included | 100.00 | Included |
| Adenosine Deaminase | ADA | INF I | 100.00 | Included | 100.00 | Included |
| CD166 antigen | ALCAM | CVD III | 100.00 | Included | 100.00 | Included |
| Tyrosine-protein kinase receptor UFO | AXL | CVD III | 100.00 | Included | 100.00 | Included |
| Beta-nerve growth factor | Beta-NGF | NEU I, INF I | 100.00 | Included | 100.00 | Included |
| C-C motif chemokine 15 | CCL15 | CVD III | 100.00 | Included | 100.00 | Included |
| C-C motif chemokine 19 | CCL19 | INF I | 100.00 | Included | 100.00 | Included |
| C-C motif chemokine 23 | CCL23 | INF I | 100.00 | Included | 100.00 | Included |
| C-C motif chemokine 3 | CCL3 | INF I | 100.00 | Included | 100.00 | Included |
| C-C motif chemokine 4 | CCL4 | INF I | 100.00 | Included | 100.00 | Included |
| Scavenger receptor cysteine-rich type 1 protein M130 | CD163 | CVD III | 100.00 | Included | 100.00 | Included |
| CD40L receptor | CD40 | INF I | 100.00 | Included | 100.00 | Included |
| CUB domain-containing protein 1 | CDCP1 | INF I | 100.00 | Included | 100.00 | Included |
| Chitinase-3-like protein 1 | CHI3L1 (YKL-40) | CVD III | 100.00 | Included | 100.00 | Included |
| Contactin-1 | CNTN1 | CVD III | 100.00 | Included | 100.00 | Included |
| Collagen alpha-1I chain | COL1A1 | CVD III | 100.00 | Included | 100.00 | Included |
| Carboxypeptidase B | CPB1 | CVD III | 100.00 | Included | 100.00 | Included |
| Macrophage colony-stimulating factor 1 | CSF-1 | INF I | 100.00 | Included | 100.00 | Included |
| Cystatin D | CST5 | INF I | 100.00 | Included | 100.00 | Included |
| Cathepsin D | CTSD | CVD III | 100.00 | Included | 100.00 | Included |
| Cathepsin Z | CTSZ | CVD III | 100.00 | Included | 100.00 | Included |
| Fractalkine | CX3CL1 | INF I | 100.00 | Included | 100.00 | Included |
| C-X-C motif chemokine 1 | CXCL1 | INF I | 100.00 | Included | 100.00 | Included |
| C-X-C motif chemokine 10 | CXCL10 | INF I | 100.00 | Included | 100.00 | Included |
| C-X-C motif chemokine 11 | CXCL11 | INF I | 100.00 | Included | 100.00 | Included |
| C-X-C motif chemokine 16 | CXCL16 | CVD III | 100.00 | Included | 100.00 | Included |
| C-X-C motif chemokine 5 | CXCL5 | INF I | 100.00 | Included | 100.00 | Included |
| C-X-C motif chemokine 6 | CXCL6 | INF I | 100.00 | Included | 100.00 | Included |
| C-X-C motif chemokine 9 | CXCL9 | INF I | 100.00 | Included | 100.00 | Included |
| Protein delta homolog 1 | DLK-1 | CVD III | 100.00 | Included | 100.00 | Included |
| Delta and Notch-like epidermal growth factor- related receptor | DNER | INF I | 100.00 | Included | 100.00 | Included |
| Epidermal growth factor receptor | EGFR | CVD III | 100.00 | Included | 100.00 | Included |
| Tumor necrosis factor receptor superfamily member 6 | FAS | CVD III | 100.00 | Included | 100.00 | Included |
| Fibroblast growth factor 19 | FGF-19 | INF I | 100.00 | Included | 100.00 | Included |
| Fms-related tyrosine kinase 3 ligand | Flt3L | INF I | 100.00 | Included | 100.00 | Included |
| Galectin-3 | Gal-3 | CVD III | 100.00 | Included | 100.00 | Included |
| Growth/differentiation factor 15 | GDF-15 | CVD III | 100.00 | Included | 100.00 | Included |
| Granulins | GRN | CVD III | 100.00 | Included | 100.00 | Included |
| Hepatocyte growth factor | HGF | INF I | 100.00 | Included | 100.00 | Included |
| Insulin-like growth factor-binding protein 2 | IGFBP-2 | CVD III | 100.00 | Included | 100.00 | Included |
| Insulin-like growth factor-binding protein 7 | IGFBP-7 | CVD III | 100.00 | Included | 100.00 | Included |
| Interleukin-10 receptor subunit beta | IL-10RB | INF I | 100.00 | Included | 100.00 | Included |
| Interleukin-12 subunit alpha | IL-12A | NEU I | 100.00 | Included | 100.00 | Included |
| Interleukin-18-binding protein | Cd38BP | CVD III | 100.00 | Included | 100.00 | Included |
| Interleukin-18 receptor 1 | IL-18R1 | INF I | 100.00 | Included | 100.00 | Included |
| Interleukin-1 receptor type 1 | IL-1RT1 | CVD III | 100.00 | Included | 100.00 | Included |
| Interleukin-12 subunit alpha | IL12A | INF, NEU | 100.00 | Included | 100.00 | Included |
| Interleukin-18 | IL18 | INF I | 100.00 | Included | 100.00 | Included |
| Interleukin-6 | IL6 | INF I | 100.00 | Included | 100.00 | Included |
| Interleukin-8 | IL8 | INF I | 100.00 | Included | 100.00 | Included |
| Latency-associated peptide transforming growth factor beta-1 | LAP TGF-beta-1 | INF I | 100.00 | Included | 100.00 | Included |
| Leukemia inhibitory factor receptor | LIF-R | INF I | 100.00 | Included | 100.00 | Included |
| Lymphotoxin-beta receptor | LTBR | CVD III | 100.00 | Included | 100.00 | Included |
| Monocyte chemotactic protein 1 | MCP-1 | CVD III, INF I | 100.00 | Included | 100.00 | Included |
| Monocyte chemotactic protein 2 | MCP-2 | INF I | 100.00 | Included | 100.00 | Included |
| Osteoprotegerin | OPG | CVD III, INF I | 100.00 | Included | 100.00 | Included |
| Osteopontin | OPN | CVD III | 100.00 | Included | 100.00 | Included |
| Plasminogen activator inhibitor 1 | PAI | CVD III | 100.00 | Included | 100.00 | Included |
| Programmed cell death 1 ligand 1 | PD-L1 | INF I | 100.00 | Included | 100.00 | Included |
| Perlecan | PLC | CVD III | 100.00 | Included | 100.00 | Included |
| Retinoic acid receptor responder protein 2 | RARRES2 | CVD III | 100.00 | Included | 100.00 | Included |
| Stem cell factor | SCF | INF I | 100.00 | Included | 100.00 | Included |
| Tyrosine-protein phosphatase non-receptor type substrate 1 | SHPS-1 | CVD III | 100.00 | Included | 100.00 | Included |
| Trefoil factor 3 | TFF3 | CVD III | 100.00 | Included | 100.00 | Included |
| Tissue factor pathway inhibitor | TFPI | CVD III | 100.00 | Included | 100.00 | Included |
| Transforming growth factor alpha | TGF-alpha | INF I | 100.00 | Included | 100.00 | Included |
| Metalloproteinase inhibitor 4 | TIMP4 | CVD III | 100.00 | Included | 100.00 | Included |
| Tumor necrosis factor receptor 1 | TNF-R1 | CVD III | 100.00 | Included | 100.00 | Included |
| Tumor necrosis factor receptor 2 | TNF-R2 | CVD III | 100.00 | Included | 100.00 | Included |
| Tumor necrosis factor receptor superfamily member 14 | TNFRSF14 | CVD III | 100.00 | Included | 100.00 | Included |
| Tumor necrosis factor receptor superfamily member 9 | TNFRSF9 | INF I | 100.00 | Included | 100.00 | Included |
| Tumor necrosis factor ligand superfamily member 13B | TNFSF13B | CVD III | 100.00 | Included | 100.00 | Included |
| TNF-related apoptosis-inducing ligand | TRAIL | INF I | 100.00 | Included | 100.00 | Included |
| Tumor necrosis factor Ligand superfamily, member 12 | TWEAK | INF I | 100.00 | Included | 100.00 | Included |
| Urokinase plasminogen activator surface receptor | U-PAR | CVD III | 100.00 | Included | 100.00 | Included |
| Urokinase-type plasminogen activator | uPA | CVD III, INF I | 100.00 | Included | 100.00 | Included |
| Vascular endothelial growth factor A | VEGFA | INF I | 100.00 | Included | 100.00 | Included |
| Interleukin-6 receptor subunit alpha | IL-6RA | CVD III | 100.00 | Included | 99.94 | Included |
| Disintegrin and metalloproteinase domain- containing protein 22 | ADAM 22 | NEU I | 100.00 | Included | 99.81 | Included |
| Disintegrin and metalloproteinase domain- containing protein 23 | ADAM 23 | NEU I | 100.00 | Included | 99.81 | Included |
| Alpha-2-macroglobulin receptor-associated protein | Alpha-2-MRAP | NEU I | 100.00 | Included | 99.81 | Included |
| Brevican core protein | BCAN | NEU I | 100.00 | Included | 99.81 | Included |
| Cell adhesion molecule 3 | CADM3 | NEU I | 100.00 | Included | 99.81 | Included |
| OX-2 membrane glycoprotein | CD200 | NEU I | 100.00 | Included | 99.81 | Included |
| ADP-ribosyl cyclase/cyclic ADP-ribose hydrolase 1 | CD38 | NEU I | 100.00 | Included | 99.81 | Included |
| Cadherin-3 | CDH3 | NEU I | 100.00 | Included | 99.81 | Included |
| Cadherin-6 | CDH6 | NEU I | 100.00 | Included | 99.81 | Included |
| CMRF35-like molecule 1 | CLM-1 | NEU I | 100.00 | Included | 99.81 | Included |
| CMRF35-like molecule 6 | CLM-6 | NEU I | 100.00 | Included | 99.81 | Included |
| Contactin-5 | CNTN5 | NEU I | 100.00 | Included | 99.81 | Included |
| Carboxypeptidase A2 | CPA2 | NEU I | 100.00 | Included | 99.81 | Included |
| Carboxypeptidase M | CPM | NEU I | 100.00 | Included | 99.81 | Included |
| Cathepsin S | CTSS | NEU I | 100.00 | Included | 99.81 | Included |
| Epithelial discoidin domain-containing receptor 1 | DDR1 | NEU I | 100.00 | Included | 99.81 | Included |
| Dickkopf-related protein 4 | Dkk-4 | NEU I | 100.00 | Included | 99.81 | Included |
| Draxin | DRAXIN | NEU I | 100.00 | Included | 99.81 | Included |
| Tumor necrosis factor receptor superfamily member 27 | EDA2R | NEU I | 100.00 | Included | 99.81 | Included |
| Ephrin-A4 | EFNA4 | NEU I | 100.00 | Included | 99.81 | Included |
| Ephrin type-B receptor 6 | EPHB6 | NEU I | 100.00 | Included | 99.81 | Included |
| Ezrin | EZR | NEU I | 100.00 | Included | 99.81 | Included |
| Leucine-rich repeat transmembrane protein FLRT2 | FLRT2 | NEU I | 100.00 | Included | 99.81 | Included |
| Galectin-8 | gal-8 | NEU I | 100.00 | Included | 99.81 | Included |
| Glypican-5 | GCP5 | NEU I | 100.00 | Included | 99.81 | Included |
| Growth/differentiation factor 8 | GDF-8 | NEU I | 100.00 | Included | 99.81 | Included |
| GDNF family receptor alpha-3 | GDNFR-alpha-3 | NEU I | 100.00 | Included | 99.81 | Included |
| GDNF family receptor alpha-1 | GFR-alpha-1 | NEU I | 100.00 | Included | 99.81 | Included |
| Granulocyte-macrophage colony-stimulating factor receptor subunit alpha | GM-CSF-R-alpha | NEU I | 100.00 | Included | 99.81 | Included |
| Granzyme A | GZMA | NEU I | 100.00 | Included | 99.81 | Included |
| Junctional adhesion molecule B | JAM-B | NEU I | 100.00 | Included | 99.81 | Included |
| Kynureninase | KYNU | NEU I | 100.00 | Included | 99.81 | Included |
| Layilin | LAYN | NEU I | 100.00 | Included | 99.81 | Included |
| Mesencephalic astrocyte-derived neurotrophic factor | MANF | NEU I | 100.00 | Included | 99.81 | Included |
| Matrilin-3 | MATN3 | NEU I | 100.00 | Included | 99.81 | Included |
| MAM domain-containing glycosylphosphatidylinositol anchor protein 1 | MDGA1 | NEU I | 100.00 | Included | 99.81 | Included |
| Macrophage scavenger receptor types I and II | MSR1 | NEU I | 100.00 | Included | 99.81 | Included |
| NKG2D ligand 2 | N2DL-2 | NEU I | 100.00 | Included | 99.81 | Included |
| N-acylethanolamine-hydrolyzing acid amidase | NAAA | NEU I | 100.00 | Included | 99.81 | Included |
| Neuroblastoma suppressor of tumorigenicity 1 | NBL1 | NEU I | 100.00 | Included | 99.81 | Included |
| Neurocan core protein | NCAN | NEU I | 100.00 | Included | 99.81 | Included |
| Neuronal cell adhesion molecule | Nr-CAM | NEU I | 100.00 | Included | 99.81 | Included |
| BDNF/NT-3 growth factors receptor | NTRK2 | NEU I | 100.00 | Included | 99.81 | Included |
| NT-3 growth factor receptor | NTRK3 | NEU I | 100.00 | Included | 99.81 | Included |
| Platelet-derived growth factor receptor alpha | PDGF-R-alpha | NEU I | 100.00 | Included | 99.81 | Included |
| Plexin-B1 | PLXNB1 | NEU I | 100.00 | Included | 99.81 | Included |
| Protogenin | PRTG | NEU I | 100.00 | Included | 99.81 | Included |
| Poliovirus receptor | PVR | NEU I | 100.00 | Included | 99.81 | Included |
| RGM domain family member B | RGMB | NEU I | 100.00 | Included | 99.81 | Included |
| Roundabout homolog 2 | ROBO2 | NEU I | 100.00 | Included | 99.81 | Included |
| Scavenger receptor class A member 5 | SCARA5 | NEU I | 100.00 | Included | 99.81 | Included |
| Lysosome membrane protein 2 | SCARB2 | NEU I | 100.00 | Included | 99.81 | Included |
| Scavenger receptor class F member 2 | SCARF2 | NEU I | 100.00 | Included | 99.81 | Included |
| Secreted frizzled-related protein 3 | sFRP-3 | NEU I | 100.00 | Included | 99.81 | Included |
| Sialic acid-binding Ig-like lectin 9 | Siglec-9 | NEU I | 100.00 | Included | 99.81 | Included |
| Serine/threonine-protein kinase receptor R3 | SKR3 | NEU I | 100.00 | Included | 99.81 | Included |
| SPARC-related modular calcium-binding protein 2 | SMOC2 | NEU I | 100.00 | Included | 99.81 | Included |
| Sphingomyelin phosphodiesterase | SMPD1 | NEU I | 100.00 | Included | 99.81 | Included |
| Testican-1 | SPOCK1 | NEU I | 100.00 | Included | 99.81 | Included |
| Rgmb  -1 membrane glycoprotein | THY 1 | NEU I | 100.00 | Included | 99.81 | Included |
| Transmembrane protease serine 5 | TMPRSS5 | NEU I | 100.00 | Included | 99.81 | Included |
| Tenascin-R | TN-R | NEU I | 100.00 | Included | 99.81 | Included |
| Tumor necrosis factor receptor superfamily member 12A | TNFRSF12A | NEU I | 100.00 | Included | 99.81 | Included |
| Tumor necrosis factor receptor superfamily member 21 | TNFRSF21 | NEU I | 100.00 | Included | 99.81 | Included |
| Netrin receptor UNC5C | UNC5C | NEU I | 100.00 | Included | 99.81 | Included |
| Brorin | VWC2 | NEU I | 100.00 | Included | 99.81 | Included |
| Bone morphogenetic protein 4 | BMP-4 | NEU I | 100.00 | Included | 99.75 | Included |
| Dipeptidyl peptidase 1 | CTSC | NEU I | 100.00 | Included | 99.75 | Included |
| Repulsive guidance molecule A | RGMA | NEU I | 100.00 | Included | 99.75 | Included |
| Plexin-B3 | PLXNB3 | NEU I | 100.00 | Included | 99.69 | Included |
| Interleukin-12 subunit beta | IL-12B | NEU I, INF I | 99.94 | Included | 100.00 | Included |
| Matrix metalloproteinase-1 | u-1 | INF I | 99.94 | Included | 100.00 | Included |
| Matrix metalloproteinase-10 | MMP-10 | INF I | 99.94 | Included | 100.00 | Included |
| Complement component C1q receptor | CD93 | CVD III | 99.94 | Included | 100.00 | Included |
| Matrix metalloproteinase-2 | MMP-2 | CVD III | 99.94 | Included | 100.00 | Included |
| Neuropilin-2 | NRP2 | NEU I | 99.94 | Included | 99.81 | Included |
| Sialoadhesin | SIGLEC1 | NEU I | 99.94 | Included | 99.81 | Included |
| Natural killer cell receptor 2B4 | CD244 | INF I | 99.88 | Included | 100.00 | Included |
| Eotaxin-1 | CCL11 | INF I | 99.81 | Included | 100.00 | Included |
| T-cell surface glycoprotein CD5 | CD5 | INF I | 99.81 | Included | 100.00 | Included |
| C-type lectin domain family 1 member B | CLEC1B | NEU I | 99.75 | Included | 99.81 | Included |
| Neprilysin | NEP | NEU I | 99.75 | Included | 99.81 | Included |
| Cell surface glycoprotein CD200 receptor 1 | CD200R1 | NEU I | 99.56 | Included | 99.81 | Included |
| Leukocyte-associated immunoglobulin-like receptor 2 | LAIR-2 | NEU I | 99.50 | Included | 98.82 | Included |
| C-C motif chemokine 25 | CCL25 | INF I | 99.38 | Included | 100.00 | Included |
| Neutral ceramidase | N-CDase | NEU I | 99.32 | Included | 99.81 | Included |
| Interleukin-7 | IL7 | INF I | 99.19 | Included | 100.00 | Included |
| Low-density lipoprotein receptor | LDL receptor | CVD III | 99.13 | Included | 100.00 | Included |
| R-spondin-1 | RSPO1 | NEU I | 99.00 | Included | 99.75 | Included |
| Matrix metalloproteinase-3 | MMP-3 | CVD III | 98.82 | Included | 100.00 | Included |
| SIR2-like protein 2 | SIRT2 | INF I | 98.38 | Included | 98.38 | Included |
| Kallikrein-6 | KLK6 | CVD III | 98.00 | Included | 100.00 | Included |
| Tumor necrosis factor ligand superfamily member 14 | TNFSF14 | INF I | 98.00 | Included | 100.00 | Included |
| Tissue-type plasminogen activator | t-PA | CVD III | 97.95 | Included | 100.00 | Included |
| C-C motif chemokine 28 | CCL28 | INF I | 97.89 | Included | 100.00 | Included |
| Fibroblast growth factor 5 | FGF-5 | INF I | 97.89 | Included | 97.76 | Included |
| N-terminal prohormone brain natriuretic peptide | NT-proBNP | CVD III | 97.88 | Included | 99.44 | Included |
| Platelet-derived growth factor subunit A | PDGF subunit A | CVD III | 97.45 | Included | 100.00 | Included |
| Neurogenic locus notch homolog protein 3 | Notch 3 | CVD III | 97.01 | Included | 100.00 | Included |
| Tumor necrosis factor receptor superfamily member 10C | TNFRSF10C | CVD III | 96.89 | Included | 100.00 | Included |
| TNF-beta | TNFB | INF I | 96.15 | Included | 100.00 | Included |
| Cytotoxic and regulatory T-cell molecule | CRTAM | NEU I | 96.14 | Included | 99.81 | Included |
| Chitotriosidase-1 | CHIT1 | CVD III | 94.71 | Included | 95.89 | Included |
| C-C motif chemokine 16 | CCL16 | CVD III | 93.15 | Included | 100.00 | Included |
| Cystatin-B | CSTB | CVD III | 93.09 | Included | 100.00 | Included |
| WAP, Kazal, immunoglobulin, Kunitz and NTR domain-containing protein 1 | WFIKKN1 | NEU I | 88.11 | Included | 99.81 | Included |
| Intercellular adhesion molecule 2 | ICAM-2 | CVD III | 88.05 | Included | 100.00 | Included |
| Interleukin-17 receptor A | IL-17RA | CVD III | 86.31 | Included | 100.00 | Included |
| Carboxypeptidase A1 | CPA1 | CVD III | 85.31 | Included | 100.00 | Included |
| STAM-binding protein | STAMPB | INF I | 84.21 | Included | 100.00 | Included |
| Myoglobin | MB | CVD III | 80.21 | Included | 100.00 | Included |
| P-selectin | SELP | CVD III | 79.96 | Included | 100.00 | Included |
| Insulin-like growth factor-binding protein 1 | IGFBP-1 | CVD III | 79.09 | Included | 100.00 | Included |
| Fibroblast growth factor 21 | FGF-21 | INF I | 76.44 | Included | 100.00 | Included |
| Nicotinamide/nicotinic acid mononucleotide adenylyltransferase 1 | NMNAT1 | NEU I | 76.04 | Included | 99.81 | Included |
| Caspase-8 | CASP-8 | INF I | 72.00 | Included | 100.00 | Included |
| E-selectin | SELE | CVD III | 71.44 | Included | 100.00 | Included |
| Tartrate-resistant acid phosphatase type 5 | TR-AP | CVD III | 67.39 | Excluded | 100.00 | Included |
| Platelet endothelial cell adhesion molecule | PECAM-1 | CVD III | 64.59 | Excluded | 100.00 | Included |
| Peptidoglycan recognition protein 1 | PGLYRP1 | CVD III | 62.35 | Excluded | 100.00 | Included |
| Aminopeptidase N | AP-N | CVD III | 60.80 | Excluded | 100.00 | Included |
| Interleukin-5 receptor subunit alpha | IL-5R-alpha | NEU I | 55.20 | Excluded | 99.81 | Included |
| Oncostatin-M | OSM | INF I | 50.47 | Excluded | 100.00 | Included |
| Leukemia inhibitory factor | LIF | INF I | 45.80 | Excluded | 18.23 | Excluded |
| Junctional adhesion molecule A | JAM-A | CVD III | 44.56 | Excluded | 100.00 | Included |
| Transferrin receptor protein 1 | TR | CVD III | 43.81 | Excluded | 100.00 | Included |
| Hydroxyacylglutathione hydrolase, mitochondrial | HAGH | NEU I | 38.02 | Excluded | 97.57 | Included |
| Fatty acid-binding protein, adipocyte | FABP4 | CVD III | 32.98 | Excluded | 100.00 | Included |
| Spondin-1 | SPON1 | CVD III | 30.62 | Excluded | 88.11 | Included |
| Fibroblast growth factor 23 | FGF-23 | INF I | 29.71 | Excluded | 100.00 | Included |
| Resistin | RETN | CVD III | 26.38 | Excluded | 100.00 | Included |
| Bleomycin hydrolase | BLM hydrolase | CVD III | 25.39 | Excluded | 100.00 | Included |
| Glial cell line-derived neurotrophic factor | GDNF | NEU I, INF I | 19.79 | Excluded | 99.75 | Included |
| Integrin beta-2 | ITGB2 | CVD III | 17.17 | Excluded | 100.00 | Included |
| C-C motif chemokine 20 | CCL20 | INF I | 14.92 | Excluded | 100.00 | Included |
| Ephrin type-B receptor 4 | EPHB4 | CVD III | 14.87 | Excluded | 100.00 | Included |
| Interleukin-1 receptor type 2 | IL-1RT2 | CVD III | 14.87 | Excluded | 100.00 | Included |
| Epithelial cell adhesion molecule | Ep-CAM | CVD III | 14.75 | Excluded | 100.00 | Included |
| Latexin | LXN | NEU I | 13.57 | Excluded | 99.81 | Included |
| ST2 protein | ST2 | CVD III | 12.69 | Excluded | 100.00 | Included |
| Caspase-3 | CASP-3 | CVD III | 11.89 | Excluded | 100.00 | Included |
| Elafin | PI3 | CVD III | 11.89 | Excluded | 99.94 | Included |
| C-C motif chemokine 24 | CCL24 | CVD III | 10.70 | Excluded | 100.00 | Included |
| Interleukin-2 receptor subunit alpha | IL2-RA | CVD III | 9.96 | Excluded | 100.00 | Included |
| C-type lectin domain family 10 member A | CLEC10A | NEU I | 8.40 | Excluded | 99.81 | Included |
| Monocyte chemotactic protein 4 | MCP-4 | INF I | 8.20 | Excluded | 100.00 | Included |
| Interleukin-10 | IL10 | INF I | 7.96 | Excluded | 100.00 | Included |
| Proprotein convertase subtilisin/kexin type 9 | PCSK9 | CVD III | 7.53 | Excluded | 100.00 | Included |
| Monocyte chemotactic protein 3 | MCP-3 | INF I | 7.52 | Excluded | 99.07 | Included |
| Secretoglobin family 3A member 2 | SCGB3A2 | CVD III | 7.09 | Excluded | 100.00 | Included |
| Matrix extracellular phosphoglycoprotein | MEPE | CVD III | 6.04 | Excluded | 100.00 | Included |
| Galectin-4 | Gal-4 | CVD III | 5.29 | Excluded | 100.00 | Included |
| Paraoxonase | PON3 | CVD III | 4.98 | Excluded | 100.00 | Included |
| Granulocyte Colony-Stimulating Factor | G-CSF | NEU I | 4.73 | Excluded | 99.81 | Included |
| von Willebrand factor | vWF | CVD III | 4.42 | Excluded | 100.00 | Included |
| Interleukin-10 receptor subunit alpha | IL-10RA | INF I | 4.29 | Excluded | 93.28 | Included |
| Protein S100-A12 | EN-RAGE | INF I | 4.00 | Excluded | 100.00 | Included |
| Signaling lymphocytic activation molecule | SLAMF1 | INF I | 3.73 | Excluded | 99.88 | Included |
| Fc receptor-like protein 2 | FcRL2 | NEU I | 3.61 | Excluded | 99.81 | Included |
| Matrix metalloproteinase-9 | MMP-9 | CVD III | 3.24 | Excluded | 100.00 | Included |
| Myeloperoxidase | MPO | CVD III | 3.05 | Excluded | 100.00 | Included |
| Interleukin-20 receptor subunit alpha | IL-20RA | INF I | 2.92 | Excluded | 18.54 | Excluded |
| T cell surface glycoprotein CD6 isoform | CD6 | INF I | 2.73 | Excluded | 100.00 | Included |
| Artemin | ARTN | INF I | 2.67 | Excluded | 23.40 | Excluded |
| Neurotrophin-3 | NT-3 | INF I | 2.61 | Excluded | 99.94 | Included |
| TNF-related activation-induced cytokine | TRANCE | INF I | 2.55 | Excluded | 100.00 | Included |
| Interleukin-5 | IL5 | INF I | 2.49 | Excluded | 44.68 | Excluded |
| Cadherin-5 | CDH5 | CVD III | 2.43 | Excluded | 100.00 | Included |
| Linker for activation of T-cells family member 1 | LAT | NEU I | 2.43 | Excluded | 99.81 | Included |
| Interleukin-17C | IL-17C | INF I | 2.42 | Excluded | 97.45 | Included |
| Trem-like transcript 2 protein | TLT-2 | CVD III | 2.36 | Excluded | 100.00 | Included |
| Pulmonary surfactant-associated protein D | PSP-D | CVD III | 2.30 | Excluded | 93.65 | Included |
| Myeloblastin | PRTN3 | CVD III | 2.24 | Excluded | 100.00 | Included |
| Azurocidin | AZU1 | CVD III | 2.18 | Excluded | 99.32 | Included |
| Interleukin-4 | IL4 | INF I | 2.05 | Excluded | 12.07 | Excluded |
| Axin-1 | AXIN1 | INF I | 1.86 | Excluded | 98.01 | Included |
| Interleukin-17A | IL-17A | INF I | 0.75 | Excluded | 72.43 | Included |
| Thymic stromal lymphopoietin | TSLP | INF I | 0.68 | Excluded | 5.48 | Excluded |
| Neurturin | NRTN | INF I | 0.37 | Excluded | 19.91 | Excluded |
| Interleukin-13 | IL13 | INF I | 0.31 | Excluded | 21.28 | Excluded |
| Interleukin-1 alpha | IL-1 alpha | INF I | 0.31 | Excluded | 0.37 | Excluded |
| Interleukin-24 | IL-24 | INF I | 0.25 | Excluded | 14.19 | Excluded |
| Sulfotransferase 1A1 | ST1A1 | INF I | 0.19 | Excluded | 97.76 | Included |
| Interleukin-15 receptor subunit alpha | IL-15RA | INF I | 0.19 | Excluded | 64.65 | Excluded |
| Interleukin-20 | IL-20 | INF I | 0.19 | Excluded | 17.61 | Excluded |
| Interleukin-2 receptor subunit beta | IL-2RB | INF I | 0.19 | Excluded | 15.12 | Excluded |
| Tumor necrosis factor | TNF | INF I | 0.12 | Excluded | 22.34 | Excluded |
| Interleukin-33 | IL33 | INF I | 0.12 | Excluded | 7.47 | Excluded |
| Interferon gamma | IFN-gamma | INF I | 0.06 | Excluded | 7.22 | Excluded |
| Interleukin-2 | IL2 | INF I | 0.06 | Excluded | 3.17 | Excluded |
| Interleukin-22 receptor subunit alpha-1 | IL-22 RA1 | INF I | 0.06 | Excluded | 2.24 | Excluded |
| Brain-derived neurotrophic factor | BDNF | NEU I, INF I | 0.00 | Excluded | 0.00 | Excluded |
| C-C motif chemokine 22 | CCL22 | CVD III | 0.00 | Excluded | 0.00 | Excluded |

***Green text*** *= excluded in CSF, included in plasma*

***Red text*** *= excluded in plasma and CSF*

***INF I*** *= inflammation 1 Olink panel*

***CVD III*** *= cardiovascular III Olink panel*

***NEU I*** *= neurology Olink panel*

**Supplementary Table S2.** Cerebrospinal fluid proteins selected in multivariate LASSO analyses (main analysis, adjusted for age, gender and mean protein concentration)

| Protein/covariate | AD Aβ+ | MCI Aβ+ | CN Aβ+ |
| --- | --- | --- | --- |
| CSF_LDLreceptor_NPX | -0,1758 | NA | NA |
| CSF_IL_17RA_NPX | -0,0553 | -0,1552 | NA |
| CSF_TNF_R2_NPX | NA | 0,1166 | NA |
| CSF_OPG_NPX | 0,0168 | NA | NA |
| CSF_SELP_NPX | NA | -0,2387 | NA |
| CSF_Gal_3_NPX | NA | NA | -0,0407 |
| CSF_NT_proBNP_NPX | NA | -0,1926 | NA |
| CSF_FABP4_NPX | NA | -0,282 | NA |
| CSF_SELE_NPX | NA | NA | -0,0071 |
| CSF_CHIT1_NPX | 0,3837 | 0,5063 | 0,1453 |
| CSF_AP_N_NPX | -0,0462 | NA | NA |
| CSF_MMP_2_NPX | -0,1379 | NA | NA |
| CSF_MB_NPX | -0,0325 | -0,1369 | NA |
| CSF_OPN_NPX | 0,1603 | 0,4499 | NA |
| CSF_CTSD_NPX | NA | 0,065 | NA |
| CSF_SHPS_1_NPX | -0,0427 | NA | NA |
| CSF_CHI3L1_NPX | 0,32 | 0,2061 | 0,1862 |
| CSF_t_PA_NPX | NA | -0,024 | NA |
| CSF_IGFBP_7_NPX | -0,228 | -0,1368 | NA |
| CSF_CD93_NPX | -0,0823 | NA | NA |
| CSF_IGFBP_2_NPX | NA | NA | -0,0043 |
| CSF_IL8_NPX | NA | 0,1289 | NA |
| CSF_VEGFA_NPX | -0,5018 | -0,4991 | NA |
| CSF_IL7_NPX | NA | -0,0973 | NA |
| CSF_IL6_NPX | NA | NA | -0,0209 |
| CSF_OSM_NPX | -0,013 | NA | NA |
| CSF_CCL4_NPX | -0,0995 | -0,0704 | NA |
| CSF_FGF_23_NPX | NA | 0,1846 | NA |
| CSF_MMP_1_NPX | -0,1852 | -0,4816 | NA |
| CSF_CCL19_NPX | -0,049 | -0,1916 | -2,00E-04 |
| CSF_Beta_NGF_NPX | NA | -0,1429 | NA |
| CSF_HGF_NPX | NA | 0,5005 | NA |
| CSF_MMP_10_NPX | 1,1643 | 0,5627 | NA |
| CSF_CD5_NPX | NA | -0,3425 | NA |
| CSF_CCL3_NPX | 0,0419 | 0,0982 | NA |
| CSF_FGF_19_NPX | -0,0306 | NA | NA |
| CSF_LIF_NPX | -0,3778 | NA | NA |
| CSF_TNFRSF9_NPX | NA | -0,1985 | NA |
| CSF_STAMPB_NPX | 0,1882 | 0,1328 | 0,0166 |
| CSF_ADA_NPX | -0,0365 | -0,2024 | NA |
| CSF_NMNAT1_NPX | 0,1667 | NA | -0,0254 |
| CSF_CADM3_NPX | -0,0186 | NA | NA |
| CSF_EZR_NPX | 0,3775 | 0,2636 | NA |
| CSF_SMOC2_NPX | 0,2468 | 0,9511 | 0,3492 |
| CSF_CRTAM_NPX | NA | NA | -0,0088 |
| CSF_CD38_NPX | NA | -0,1463 | 0 |
| CSF_MSR1_NPX | NA | NA | -0,1064 |
| CSF_RGMB_NPX | NA | -0,2777 | NA |
| CSF_ADAM22_NPX | 0,2507 | NA | NA |
| CSF_RSPO1_NPX | 0 | -0,1118 | NA |
| CSF_HAGH_NPX | 0,9005 | 1,0503 | NA |
| CSF_WFIKKN1_NPX | -0,1821 | NA | NA |
| CSF_TMPRSS5_NPX | 0,0862 | NA | NA |
| CSF_CDH3_NPX | NA | -0,4109 | -0,1542 |
| CSF_GFR_alpha_1_NPX | NA | 0,2474 | NA |
| CSF_CD200_NPX | -0,4483 | -0,0015 | NA |
| CSF_GCP5_NPX | NA | NA | 0,0707 |
| CSF_PDGF_R_alpha_NPX | NA | -0,0478 | NA |
| CSF_CTSC_NPX | 0,0151 | NA | NA |
| CSF_CTSS_NPX | NA | -0,0914 | NA |
| CSF_N2DL_2_NPX | -0,1805 | -0,5743 | NA |
| CSF_CLM_1_NPX | NA | 0,0582 | NA |
| CSF_IL12_NPX | NA | -0,114 | NA |
| CSF_Dkk_4_NPX | NA | -0,1836 | NA |
| CSF_EDA2R_NPX | -0,0656 | NA | NA |
| CSF_TN_R_NPX | NA | 0,1394 | NA |
| CSF_CD200R1_NPX | -0,0036 | NA | NA |
| *Gender [Male]* | *NA* | *0,4893* | *-0,0921* |

The table shows β-coefficients from three different LASSO models, to predict each diagnostic group (AD Aβ+, MCI Aβ+, and CN A+) versus CN Aβ- using CSF proteins. The MCI Aβ- model was not significant and is therefore not shown. The LASSO selected predictors and assigned weights to selected predictors. Only features that were selected in at least one model are shown here. All biomarkers were centered and scaled for these analyses. The models were covaried for age, gender sex, and overall mean CSF protein concentrations. Gender was the only covariate that was selected, and is shown, in italics at the bottom of the table.

**Supplementary Table S3.** Plasma proteins selected in multivariate LASSO analyses (main analysis, adjusted for age, gender and mean protein concentration)

| Protein/covariate | AD Aβ+ | MCI Aβ+ |
| --- | --- | --- |
| Plasma_MMP_9_NPX | NA | -0,1554 |
| Plasma_IL2_RA_NPX | -0,2238 | NA |
| Plasma_CSTB_NPX | 0,1161 | 0,0136 |
| Plasma_GRN_NPX | 0,0921 | NA |
| Plasma_NT_proBNP_NPX | 0,0868 | NA |
| Plasma_Notch3_NPX | 0,0432 | NA |
| Plasma_TIMP4_NPX | 0,3668 | 0,1006 |
| Plasma_CCL24_NPX | -0,0103 | NA |
| Plasma_TR_NPX | 0,9695 | 0,0768 |
| Plasma_GDF_15_NPX | NA | 0,2866 |
| Plasma_SELE_NPX | -0,1084 | -0,0628 |
| Plasma_AZU1_NPX | NA | 0,1658 |
| Plasma_DLK_1_NPX | -0,0916 | -0,0205 |
| Plasma_SPON1_NPX | 0,0869 | NA |
| Plasma_MPO_NPX | 0,1807 | NA |
| Plasma_IL_6RA_NPX | NA | -0,0356 |
| Plasma_RETN_NPX | 0,0037 | NA |
| Plasma_IGFBP_1_NPX | -0,3907 | -0,0868 |
| Plasma_TR_AP_NPX | 0,2116 | NA |
| Plasma_PI3_NPX | 0,1066 | 0,3111 |
| Plasma_Ep_CAM_NPX | 0,0132 | NA |
| Plasma_MB_NPX | -0,0710 | -0,0334 |
| Plasma_TNFSF13B_NPX | -0,0323 | NA |
| Plasma_OPN_NPX | 0,2094 | 0,2080 |
| Plasma_Gal_4_NPX | -0,0172 | NA |
| Plasma_SHPS_1_NPX | -0,0786 | NA |
| Plasma_CCL15_NPX | NA | -0,0712 |
| Plasma_uPA_NPX | -0,2871 | NA |
| Plasma_CPB1_NPX | 0,2103 | NA |
| Plasma_CHI3L1_NPX | NA | 0,0111 |
| Plasma_t_PA_NPX | -0,1554 | NA |
| Plasma_EGFR_NPX | NA | -0,0305 |
| Plasma_CTSZ_NPX | NA | -0,0377 |
| Plasma_RARRES2_NPX | -0,2276 | NA |
| Plasma_ICAM_2_NPX | 0,0704 | NA |
| Plasma_KLK6_NPX | -0,4789 | NA |
| Plasma_PDGFsubunitA_NPX | NA | 0,0828 |
| Plasma_IGFBP_2_NPX | 0,1457 | 0,1363 |
| Plasma_MEPE_NPX | -0,2294 | -0,0429 |
| Plasma_CCL16_NPX | NA | -0,0884 |
| Plasma_IL8_NPX | 0,1948 | 0,0597 |
| Plasma_MCP_3_NPX | -0,1113 | NA |
| Plasma_CDCP1_NPX | -0,0701 | NA |
| Plasma_IL_17C_NPX | NA | 0,0419 |
| Plasma_CXCL11_NPX | NA | 0,0331 |
| Plasma_AXIN1_NPX | -0,2253 | NA |
| Plasma_TRAIL_NPX | NA | -0,1860 |
| Plasma_CST5_NPX | -0,1948 | -0,0274 |
| Plasma_CXCL1_NPX | 0,0744 | 0,1569 |
| Plasma_SCF_NPX | 0,3323 | 0,0965 |
| Plasma_IL18_NPX | 0,0380 | NA |
| Plasma_FGF_23_NPX | -0,0139 | NA |
| Plasma_IL_10RA_NPX | NA | -0,0441 |
| Plasma_CCL19_NPX | NA | 0,0095 |
| Plasma_IL_15RA_NPX | NA | -0,0158 |
| Plasma_IL_10RB_NPX | 0,1029 | NA |
| Plasma_IL_18R1_NPX | -0,0265 | NA |
| Plasma_TRANCE_NPX | -0,2725 | -0,0479 |
| Plasma_ARTN_NPX | 0,0845 | NA |
| Plasma_IL10_NPX | -0,0308 | NA |
| Plasma_TNF_NPX | -0,0970 | NA |
| Plasma_CCL23_NPX | 0,0144 | NA |
| Plasma_Flt3L_NPX | -0,1787 | NA |
| Plasma_4E_BP1_NPX | 0,8901 | NA |
| Plasma_EN_RAGE_NPX | 0,1372 | 0,3303 |
| Plasma_FGF_19_NPX | -0,1239 | NA |
| Plasma_CX3CL1_NPX | 0,4377 | NA |
| Plasma_IL5_NPX | -0,1149 | NA |
| Plasma_ADA_NPX | 0,0447 | NA |
| Plasma_VWC2_NPX | NA | -0,0938 |
| Plasma_SMOC2_NPX | -0,4113 | NA |
| Plasma_SCARB2_NPX | 0,0073 | NA |
| Plasma_NCAN_NPX | NA | 0,1196 |
| Plasma_PRTG_NPX | NA | 0,0654 |
| Plasma_CRTAM_NPX | 0,0319 | NA |
| Plasma_RGMA_NPX | NA | 0,0580 |
| Plasma_CD38_NPX | NA | -0,0289 |
| Plasma_SMPD1_NPX | 0,2925 | NA |
| Plasma_EPHB6_NPX | 0,0207 | NA |
| Plasma_MATN3_NPX | NA | 0,0892 |
| Plasma_RSPO1_NPX | 0,8796 | -0,0910 |
| Plasma_HAGH_NPX | NA | 0,6408 |
| Plasma_BCAN_NPX | -0,2077 | -0,0982 |
| Plasma_THY1_NPX | -0,2581 | -0,5991 |
| Plasma_TMPRSS5_NPX | 0,0308 | NA |
| Plasma_GFR_alpha_1_NPX | 0,0156 | 0,1007 |
| Plasma_GM_CSF_R_alpha_NPX | 0,0018 | NA |
| Plasma_SCARF2_NPX | 0,0761 | 0,2582 |
| Plasma_PVR_NPX | 0,0071 | 0,0500 |
| Plasma_TNFRSF12A_NPX | 0,1114 | NA |
| Plasma_BMP_4_NPX | 0,3843 | NA |
| Plasma_FcRL2_NPX | -0,0091 | NA |
| Plasma_IL_5R_alpha_NPX | 0,0440 | 0,0465 |
| Plasma_CTSC_NPX | -0,2464 | NA |
| Plasma_JAM_B_NPX | NA | -0,1946 |
| Plasma_CTSS_NPX | NA | -0,0409 |
| Plasma_N2DL_2_NPX | -0,0142 | NA |
| Plasma_PLXNB1_NPX | NA | -0,0644 |
| Plasma_SPOCK1_NPX | 0,0733 | NA |
| Plasma_LAT_NPX | NA | 0,0775 |
| Plasma_NTRK3_NPX | NA | 0,1891 |
| Plasma_TN_R_NPX | NA | -0,2449 |
| Plasma_KYNU_NPX | -0,0600 | NA |
| *Age* | *0,0028* | *-0,0153* |

The data are coefficients from two different LASSO models, to predict each diagnostic group (AD Aβ+ and MCI Aβ+) versus CN Aβ- using plasma proteins. The CN Aβ+ and MCI Aβ- models were non-significant, and coefficients from those model are therefore not shown. The LASSO selected predictors and assigned weights to the selected predictors. Only features that were selected in at least one model are shown here. All biomarkers were centered and scaled for these analyses. The models were covaried for age, sex, and overall mean plasma protein concentrations. Age was the only covariate that was selected, and is shown, in italics, at the bottom of the table.

**Supplementary Table S4.** Cerebrospinal fluid proteins selected in multivariate LASSO analyses (supplementary analysis, adjusting for all covariates)

| Protein/covariate | AD Aβ+ | MCI Aβ+ | MCI Aβ- | CN Aβ+ |
| --- | --- | --- | --- | --- |
| CSF_LDLreceptor_NPX | -0,4035 | 0,0000 | 0,0000 | 0,0000 |
| CSF_IL_17RA_NPX | -0,3423 | 0,0000 | 0,0000 | 0,0000 |
| CSF_TNF_R2_NPX | 0,0000 | 0,0341 | 0,0000 | 0,0000 |
| CSF_OPG_NPX | 0,2420 | 0,0000 | 0,0000 | 0,0000 |
| CSF_SELP_NPX | 0,0000 | -0,1376 | 0,0000 | 0,0000 |
| CSF_FABP4_NPX | 0,0000 | -0,4769 | 0,0000 | 0,0000 |
| CSF_PAI_NPX | 0,0000 | 0,0000 | 0,2821 | 0,0000 |
| CSF_SPON1_NPX | 0,0496 | 0,0000 | 0,0000 | 0,0000 |
| CSF_CHIT1_NPX | 0,2976 | 0,4133 | 0,0000 | 0,0531 |
| CSF_MMP_2_NPX | -0,4628 | 0,0000 | 0,0000 | 0,0000 |
| CSF_MB_NPX | -0,0877 | -0,0146 | 0,0000 | 0,0000 |
| CSF_OPN_NPX | 0,6284 | 0,7336 | 0,0000 | 0,0000 |
| CSF_SHPS_1_NPX | -0,0905 | 0,0000 | 0,0000 | 0,0000 |
| CSF_CHI3L1_NPX | 0,5865 | 0,0000 | 0,0000 | 0,2390 |
| CSF_IGFBP_7_NPX | -0,4265 | 0,0000 | 0,0000 | 0,0000 |
| CSF_CD93_NPX | -0,0985 | 0,0000 | 0,0000 | 0,0000 |
| CSF_VEGFA_NPX | -1,0500 | -0,1005 | 0,0000 | 0,0000 |
| CSF_OSM_NPX | -0,0348 | 0,0000 | 0,0000 | 0,0000 |
| CSF_CCL4_NPX | -0,3314 | 0,0000 | 0,0000 | 0,0000 |
| CSF_MMP_1_NPX | -0,1575 | -0,3410 | 0,0000 | 0,0000 |
| CSF_CCL19_NPX | -0,0630 | 0,0000 | 0,0000 | 0,0000 |
| CSF_MMP_10_NPX | 2,0123 | 0,7844 | 0,0000 | 0,0000 |
| CSF_CD5_NPX | 0,0000 | -0,6373 | 0,0000 | 0,0000 |
| CSF_CCL3_NPX | 0,1691 | 0,0000 | 0,0000 | 0,0000 |
| CSF_LIF_NPX | -0,7388 | 0,0000 | 0,0000 | 0,0000 |
| CSF_TNFRSF9_NPX | 0,0000 | -0,1648 | 0,0000 | 0,0000 |
| CSF_STAMPB_NPX | 0,8293 | 0,0000 | 0,0000 | 0,0000 |
| CSF_ADA_NPX | -0,0785 | 0,0000 | 0,0000 | 0,0000 |
| CSF_NMNAT1_NPX | 0,2241 | 0,0000 | 0,3423 | 0,0000 |
| CSF_CLM_6_NPX | 0,0310 | 0,0000 | 0,0000 | 0,0000 |
| CSF_EZR_NPX | 1,1127 | 0,0601 | 0,0000 | 0,0000 |
| CSF_SMOC2_NPX | 0,4194 | 0,7987 | 0,0000 | 0,4379 |
| CSF_CD38_NPX | 0,0000 | -0,1625 | 0,0000 | 0,0000 |
| CSF_RGMB_NPX | 0,0000 | -0,5321 | -0,2265 | 0,0000 |
| CSF_ADAM22_NPX | 0,4584 | 0,0000 | 0,0000 | 0,0000 |
| CSF_HAGH_NPX | 2,4762 | 1,2564 | 0,0000 | 0,0000 |
| CSF_WFIKKN1_NPX | -0,6807 | 0,0000 | 0,0000 | 0,0000 |
| CSF_TMPRSS5_NPX | 0,2030 | 0,0000 | 0,0000 | 0,0000 |
| CSF_CDH3_NPX | 0,0000 | -1,9018 | -0,0129 | -0,7042 |
| CSF_CD200_NPX | -1,0089 | 0,0000 | 0,0000 | 0,0000 |
| CSF_DRAXIN_NPX | 0,0000 | 0,0000 | -0,0350 | 0,0000 |
| CSF_GCP5_NPX | 0,0000 | 0,0000 | 0,0000 | 0,0205 |
| CSF_CTSC_NPX | 0,0280 | 0,0000 | 0,0000 | 0,0000 |
| CSF_N2DL_2_NPX | -0,3375 | -0,3416 | -0,0339 | 0,0000 |
| CSF_EDA2R_NPX | -0,1866 | 0,0000 | 0,0000 | 0,0000 |
| CSF_CD200R1_NPX | -0,3064 | 0,0000 | 0,0000 | 0,0000 |
| *Current smoker* | *0,0000* | *0,0000* | *0,0000* | *-0,1283* |
| *Platelet inihibitors* | *0,0000* | *0,1794* | *0,0114* | *0,0000* |
| *Antidepressants* | *0,8290* | *0,3087* | *1,1744* | *0,0000* |

The table shows β-coefficients from four different LASSO models, to predict each diagnostic group (AD Aβ+, MCI Aβ+, MCI Aβ- and CN A+) versus CN Aβ- using CSF proteins. The LASSO selected predictors and assigned weights to selected predictors. Only features that were selected in at least one model are shown here. All biomarkers were centered and scaled for these analyses. The models were covaried for age, sex, smoking status, overall mean CSF protein concentrations, and drug use at baseline (dichotomous data, for platelet inhibitors, antidepressants, anti-inflammatory, lipid lowering, hypertensive/cardioprotective). Covariates that were selected in at least one model are shown, in italics, with their assigned weights, at the bottom of the table.

**Supplementary Table S5.** Plasma proteins selected in multivariate LASSO analyses (supplementary analysis, adjusting for all covariates)

| Protein/covariate | AD Aβ+ | MCI Aβ- | CN Aβ+ |
| --- | --- | --- | --- |
| Plasma_MMP_9_NPX | NA | NA | 0,1396 |
| Plasma_IL2_RA_NPX | -0,4493 | NA | NA |
| Plasma_CSTB_NPX | 0,0994 | NA | 0,1291 |
| Plasma_Gal_3_NPX | NA | NA | -0,9910 |
| Plasma_GRN_NPX | NA | NA | 0,4883 |
| Plasma_NT_proBNP_NPX | 0,0374 | NA | 0,1959 |
| Plasma_BLMhydrolase_NPX | NA | NA | 0,1900 |
| Plasma_PLC_NPX | NA | NA | -0,1718 |
| Plasma_Notch3_NPX | 0,1390 | NA | NA |
| Plasma_TIMP4_NPX | 0,7028 | NA | NA |
| Plasma_TLT_2_NPX | 0,0008 | NA | NA |
| Plasma_FABP4_NPX | NA | NA | 0,2224 |
| Plasma_TFPI_NPX | NA | NA | 0,0020 |
| Plasma_TR_NPX | 1,4517 | NA | 0,1868 |
| Plasma_TNFRSF10C_NPX | NA | NA | 0,1062 |
| Plasma_SELE_NPX | NA | NA | -0,0742 |
| Plasma_DLK_1_NPX | -0,0379 | NA | NA |
| Plasma_SPON1_NPX | 0,3674 | NA | NA |
| Plasma_MPO_NPX | 0,3519 | NA | NA |
| Plasma_CXCL16_NPX | NA | NA | -0,2585 |
| Plasma_RETN_NPX | 0,0075 | NA | 0,4592 |
| Plasma_IGFBP_1_NPX | -0,3390 | NA | -0,1469 |
| Plasma_TR_AP_NPX | 0,5803 | NA | NA |
| Plasma_PSP_D_NPX | NA | NA | -0,0593 |
| Plasma_PI3_NPX | 0,2130 | NA | NA |
| Plasma_AP_N_NPX | NA | NA | 0,0876 |
| Plasma_FAS_NPX | NA | NA | -0,0158 |
| Plasma_MB_NPX | -0,2513 | NA | 0,3671 |
| Plasma_TNFSF13B_NPX | -0,1509 | NA | -0,1016 |
| Plasma_PRTN3_NPX | NA | NA | -0,5287 |
| Plasma_PCSK9_NPX | NA | NA | -0,0492 |
| Plasma_OPN_NPX | 0,1542 | NA | NA |
| Plasma_CTSD_NPX | NA | NA | -0,5486 |
| Plasma_PGLYRP1_NPX | 0,0477 | NA | NA |
| Plasma_CPA1_NPX | NA | NA | 0,1225 |
| Plasma_Gal_4_NPX | -0,0018 | NA | 0,0741 |
| Plasma_IL_1RT2_NPX | NA | NA | -0,2947 |
| Plasma_SHPS_1_NPX | -0,1518 | NA | 0,3093 |
| Plasma_CCL15_NPX | 0,1221 | NA | NA |
| Plasma_uPA_NPX | -0,7787 | NA | NA |
| Plasma_CPB1_NPX | 0,2081 | NA | 0,1614 |
| Plasma_CHI3L1_NPX | NA | NA | 0,1232 |
| Plasma_ST2_NPX | NA | NA | 0,2436 |
| Plasma_t_PA_NPX | -0,0678 | NA | 0,1159 |
| Plasma_EGFR_NPX | NA | NA | -0,1475 |
| Plasma_IGFBP_7_NPX | NA | NA | 0,0002 |
| Plasma_COL1A1_NPX | NA | NA | -0,1580 |
| Plasma_MMP_3_NPX | NA | NA | -0,4798 |
| Plasma_RARRES2_NPX | -1,0733 | NA | NA |
| Plasma_ICAM_2_NPX | 0,1218 | NA | NA |
| Plasma_KLK6_NPX | -0,9430 | NA | NA |
| Plasma_IGFBP_2_NPX | 0,2770 | NA | NA |
| Plasma_MEPE_NPX | -0,3392 | NA | NA |
| Plasma_CCL16_NPX | NA | NA | 0,1027 |
| Plasma_IL8_NPX | 0,2658 | NA | NA |
| Plasma_VEGFA_NPX | NA | NA | -0,1293 |
| Plasma_MCP_3_NPX | -0,0864 | NA | NA |
| Plasma_GDNF_NPX | NA | NA | -0,0502 |
| Plasma_CDCP1_NPX | -0,1440 | NA | NA |
| Plasma_IL6_NPX | NA | NA | -0,0383 |
| Plasma_AXIN1_NPX | -0,1769 | NA | NA |
| Plasma_TRAIL_NPX | NA | NA | -0,5821 |
| Plasma_CXCL9_NPX | NA | NA | -0,0105 |
| Plasma_CST5_NPX | -0,1230 | NA | NA |
| Plasma_OSM_NPX | NA | 0,1739 | NA |
| Plasma_CXCL1_NPX | 0,1014 | NA | 0,0329 |
| Plasma_CCL4_NPX | NA | NA | 0,1429 |
| Plasma_SCF_NPX | 0,9496 | NA | 0,2821 |
| Plasma_IL18_NPX | NA | NA | 0,3808 |
| Plasma_SLAMF1_NPX | NA | NA | -0,1424 |
| Plasma_CCL11_NPX | NA | NA | 0,2090 |
| Plasma_FGF_23_NPX | NA | NA | 0,1253 |
| Plasma_IL_10RA_NPX | NA | NA | 0,0122 |
| Plasma_FGF_5_NPX | NA | NA | 0,2119 |
| Plasma_FGF_21_NPX | NA | NA | -0,1349 |
| Plasma_IL_15RA_NPX | NA | NA | -0,1733 |
| Plasma_IL_10RB_NPX | 0,4092 | NA | 0,4737 |
| Plasma_IL_18R1_NPX | NA | NA | -0,0909 |
| Plasma_TRANCE_NPX | -0,4490 | NA | -0,0198 |
| Plasma_HGF_NPX | NA | NA | 0,0743 |
| Plasma_IL13_NPX | NA | NA | -0,0839 |
| Plasma_ARTN_NPX | 0,3022 | NA | 0,1470 |
| Plasma_IL10_NPX | -0,0256 | NA | 0,0855 |
| Plasma_TNF_NPX | -0,0746 | NA | 0,0501 |
| Plasma_CCL23_NPX | 0,0343 | NA | NA |
| Plasma_Flt3L_NPX | -0,4471 | NA | NA |
| Plasma_4E_BP1_NPX | 0,8202 | NA | NA |
| Plasma_DNER_NPX | NA | NA | 0,3228 |
| Plasma_EN_RAGE_NPX | 0,0634 | NA | 0,0482 |
| Plasma_FGF_19_NPX | -0,0927 | NA | -0,0238 |
| Plasma_LIF_NPX | -0,0215 | NA | NA |
| Plasma_CX3CL1_NPX | 1,1463 | NA | NA |
| Plasma_TNFRSF9_NPX | -0,1888 | NA | NA |
| Plasma_TWEAK_NPX | NA | NA | 0,0298 |
| Plasma_CCL20_NPX | -0,0017 | NA | NA |
| Plasma_IL5_NPX | -0,0685 | NA | -0,0277 |
| Plasma_NMNAT1_NPX | NA | NA | 0,0169 |
| Plasma_UNC5C_NPX | -0,3292 | NA | -0,1313 |
| Plasma_Siglec_9_NPX | NA | NA | -0,0159 |
| Plasma_CLM_6_NPX | 0,1583 | NA | 0,5115 |
| Plasma_SMOC2_NPX | -1,0945 | NA | NA |
| Plasma_NBL1_NPX | NA | NA | -0,0167 |
| Plasma_SCARB2_NPX | 0,2343 | NA | NA |
| Plasma_NCAN_NPX | NA | NA | 0,8661 |
| Plasma_CPA2_NPX | NA | NA | 0,1504 |
| Plasma_CD38_NPX | NA | NA | -0,0625 |
| Plasma_SMPD1_NPX | 0,5876 | NA | -0,2154 |
| Plasma_MSR1_NPX | NA | NA | -0,4243 |
| Plasma_sFRP_3_NPX | 0,0850 | NA | NA |
| Plasma_EPHB6_NPX | 0,1287 | NA | -0,0260 |
| Plasma_CLEC1B_NPX | NA | NA | -0,1812 |
| Plasma_ADAM23_NPX | NA | NA | -0,0513 |
| Plasma_MATN3_NPX | NA | NA | 0,0357 |
| Plasma_RSPO1_NPX | 1,0381 | NA | NA |
| Plasma_BCAN_NPX | -0,5169 | NA | -0,2798 |
| Plasma_LAYN_NPX | -0,2705 | NA | NA |
| Plasma_THY1_NPX | -0,9375 | NA | NA |
| Plasma_WFIKKN1_NPX | NA | NA | 0,3052 |
| Plasma_TMPRSS5_NPX | NA | NA | -0,0709 |
| Plasma_GM_CSF_R_alpha_NPX | 0,0084 | NA | 0,0123 |
| Plasma_SCARA5_NPX | NA | NA | -1,1347 |
| Plasma_NTRK2_NPX | 0,2955 | NA | 0,7388 |
| Plasma_SCARF2_NPX | 0,2270 | NA | NA |
| Plasma_TNFRSF12A_NPX | 0,2937 | NA | NA |
| Plasma_FLRT2_NPX | NA | NA | 0,1586 |
| Plasma_GCP5_NPX | NA | NA | 0,1060 |
| Plasma_BMP_4_NPX | 0,6660 | NA | NA |
| Plasma_FcRL2_NPX | -0,0348 | NA | 0,0252 |
| Plasma_MDGA1_NPX | NA | NA | 0,0792 |
| Plasma_IL_5R_alpha_NPX | 0,0166 | NA | NA |
| Plasma_PDGF_R_alpha_NPX | NA | NA | -0,1192 |
| Plasma_CTSC_NPX | -0,3600 | NA | -0,2650 |
| Plasma_CDH6_NPX | NA | NA | 0,4799 |
| Plasma_CTSS_NPX | 0,1161 | NA | NA |
| Plasma_N_CDase_NPX | NA | NA | 0,0653 |
| Plasma_N2DL_2_NPX | -0,0314 | NA | 0,1287 |
| Plasma_PLXNB1_NPX | NA | NA | 0,2814 |
| Plasma_SPOCK1_NPX | 0,2490 | NA | NA |
| Plasma_Dkk_4_NPX | NA | NA | -0,1210 |
| Plasma_NTRK3_NPX | NA | NA | 0,4118 |
| Plasma_TN_R_NPX | NA | NA | -0,1373 |
| Plasma_CD200R1_NPX | NA | NA | -0,3003 |
| Plasma_KYNU_NPX | -0,0878 | NA | NA |
| *Age* | *0,0074* | *NA* | *0,0024* |
| *Gender [Male]* | *NA* | *NA* | *-0,0956* |
| *Current smoker* | *NA* | *NA* | *-0,8586* |
| *Platelet inihibitors* | *0,1241* | *NA* | *NA* |
| *Antidepressants* | *1,0130* | *0,6510* | *NA* |
| *Antiinflammatory* | *-0,0758* | *NA* | *-0,1214* |
| *Hypertension/cardioprotective* | *NA* | *NA* | *0,0287* |

The data are coefficients from three different LASSO models, to predict each diagnostic group (AD Aβ+, MCI Aβ+, and CN Aβ+) versus CN Aβ- using plasma proteins. The MCI Aβ+ model was non-significant and coefficients from that model are therefore not shown. The LASSO selected predictors and assigned weights to the selected predictors. Only features that were selected in at least one model are shown here. All biomarkers were centered and scaled for these analyses. The models were covaried for age, sex, smoking status, overall mean plasma protein concentrations, and drug use at baseline (dichotomous data, for platelet inhibitors, antidepressants, anti-inflammatory, lipid lowering, hypertensive/cardioprotective). Covariates that were selected in at least one model are shown, in italics, with their assigned weights, at the bottom of the table.

|  | Control Aβ- | Control Aβ+ | MCI- Aβ- | MCI- Aβ+ | AD (Aβ+) |
| --- | --- | --- | --- | --- | --- |
| Sample size (n) | 59 | 23 | 44 | 53 | 0 |
| Sex (F/M) | 37/22 | 14/9 | 8/36 | 23/30 | N/A |
| Mean age in years (SD) | 68.4 (5.9) | 69.9 (4.5) | 69.6 (6) | 72.3 (4.2) | N/A |
| MMSE mean (SD) | 28.64 (1.3) | 28.4 (1.03) | 27.45 (2.2) | 26.53 (1.73) | N/A |
| APOE (1 or 2 e4 alleles) | 22% | 69.6% | 34.1% | 73.6% | N/A |
| Anti-inflammatory drugs | 18.64% | 13.04% | 11.36% | 9.43% | N/A |
| Platelet inhibitor drugs | 27.12% | 34.78% | 38.64% | 35.85% | N/A |
| Antidepressive drugs | 23.73% | 39.13% | 38.64% | 22.64% | N/A |
| Lipid-lowering drugs | 23.73% | 17.39% | 38.64% | 32.08% | N/A |
| Antihypertensive / cardioprotective drugs | 35.59% | 26.09% | 52.27% | 52.83% | N/A |
| Current smoker | 8.47% | 0.00% | 6.82% | 11.32% | N/A |
| Mean Aβ42 in pg/ml (SD) | 825 (219) | 345 (112) | 737 (242) | 387 (124) | N/A |
| Mean Aβ40 in pg/ml (SD) | 6229 (1989) | 5824 (1929) | 5565 (1864) | 6396 (2035) | N/A |
| Aβ42/40 ratio - log_2_ transformed (SD) | 2 (0.15) | 2.82 (0.29) | 2.02 (0.18) | 2.81 (0.27) | N/A |
| Mean total tau (SD) | 275 (64) | 516 (162) | 285 (83) | 528 (235) | N/A |
| Mean phospho-tau (SD) | 35 (11) | 97 (53) | 38 (17) | 102 (52) | N/A |

**Supplementary Table S6.** Demographic and clinical data for the Memory Lund ‘replication’ cohort.

*Supplementary Tables* ***S7-S39*** *are available as a downloadable Excel spreadsheet.*
